# Hes1 inhibitor isolated by target protein oriented natural products isolation (TPO-NAPI) of differentiation activators of neural stem cells†
†Electronic supplementary information (ESI) available. See DOI: 10.1039/c5sc03540f


**DOI:** 10.1039/c5sc03540f

**Published:** 2015-12-01

**Authors:** Midori A. Arai, Naoki Ishikawa, Mitsuha Tanaka, Kenji Uemura, Noriko Sugimitsu, Akiko Suganami, Yutaka Tamura, Takashi Koyano, Thaworn Kowithayakorn, Masami Ishibashi

**Affiliations:** a Graduate School of Pharmaceutical Sciences , Chiba University , 1-8-1 Inohana, Chuo-ku , Chiba 260-8675 , Japan . Email: midori_arai@chiba-u.jp ; Email: mish@chiba-u.jp; b Graduate School of Medicine , Chiba University , 1-8-1 Inohana, Chuo-ku , Chiba 260-8670 , Japan; c Temko Corporation , 4-27-4 Honcho, Nakano , Tokyo 164-0012 , Japan; d Faculty of Agriculture , Khon Kaen University , Khon Kaen 40002 , Thailand

## Abstract

The Hes1 dimer inhibitor, agalloside (**2**), which can accelerate the differentiation of neural stem cells is described. Six natural products, including one new natural product, which bind to Hes1 were rapidly isolated by a developed “target protein oriented natural products isolation” (TPO-NAPI) method using Hes1-immobilized beads. Of the six compounds, **2** inhibited Hes1 dimer formation at both the protein- and cellular level. Neural stem cells treated with **2** differentiated to neurons with longer neurites than cells treated with varproic acid or retinoic acid. Moreover, **2** exhibited specificity for neurons. This promotion of differentiation was supported by an increase in the mRNA expression of the proneural genes, *Mash1* and *Ngn2*, which were inhibited by Hes1.

## Abstract 2

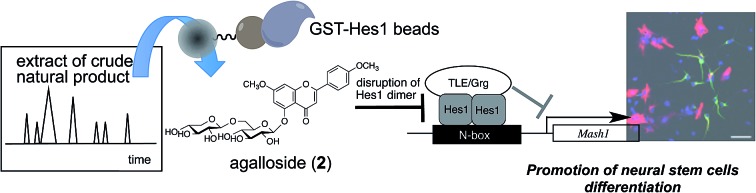
Hes1 dimer inhibitor, agalloside (**2**), which can accelerate the differentiation of neural stem cells was isolated by using Hes1-immobilized beads.

## Introduction

Neural stem cells (NSCs) can differentiate into neural cells such as neurons, astrocytes and oligodendrocytes. NSCs have been discovered in the adult mouse brain^1^ and the adult human brain (dentate gyrus,^2^ subventicular zone^3^). Endogenous or transplanted NSCs result in neurogenesis in response to injury.^4^–^6^ This discovery has led to considerable research into identifying clinical methods for using NSCs to regenerate neuronal cells damaged by stroke, spinal cord injury, or neurodegenerative disorders. Small molecules that can accelerate the differentiation of NSCs would thus be regenerative drug candidates. Varproic acid has been reported to improve the restoration of hind leg function of spinal cord injury model mice.^7^ However, although there have been many reports of neurite-growth-promoting small molecules,^8^–^12^ the number of small molecules reported to accelerate the differentiation of neural stem cells is still quite limited.^13^–^22^

Basic-helix-loop-helix (bHLH) transcription factors control the fate of neural stem cells, *i.e.*, their proliferation and differentiation.^23^–^26^ Activator-type bHLH factors such as Mash1 (also known as Ascl1), neurogenin2 (Ngn2) and NeuroD affect the differentiation of NSCs to neural cells. On the other hand, repressor-type bHLH factors, such as hairy and Enhancer of split 1 (Hes1) and Hes5, maintain NSCs in the undifferentiated form and enhance their self-proliferation. Hes1 inhibits the expression of activator-type bHLH factors by binding to the promoter region as a homo dimer to recruit the co-repressor, TLE/Grg. We postulated that small molecule inhibitors of Hes1 dimer formation would accelerate NSC differentiation.

Recent technical innovations in small molecule screening using huge small molecule libraries have led to the successful use of reverse chemical genetics using immobilized target proteins.^27^–^30^ However, in contrast to the rapid innovations in large-scale screening, innovations in “target protein oriented natural product isolation” (TPO-NAPI) have developed more slowly.^31^–^39^ We previously reported an isolation of new natural products which bind a target protein using protein-immobilized beads-HPLC method.^39^ The ability of a natural product to bind to a target protein is important for estimating the bioactivity of the natural product. Thus, a target protein-beads-HPLC method would be a powerful approach for isolating bioactive natural compounds from natural product extracts.

Here we report target protein oriented natural products isolation (TPO-NAPI) using a protein-immobilized beads-HPLC method for NSCs differentiation activators. Six natural products, including one new compound, were isolated. One of these compounds, agalloside (**2**) accelerated NSC differentiation by disrupting dimer formation of Hes1, a repressor-type bHLH transcriptional factor. To the best of our knowledge, this is the first example of the acceleration of NSC differentiation by a Hes1 dimer inhibitor.

## Results and discussion

### TPO-NAPI for naturally occuring Hes1 inhibitors

**Fig. 1:**
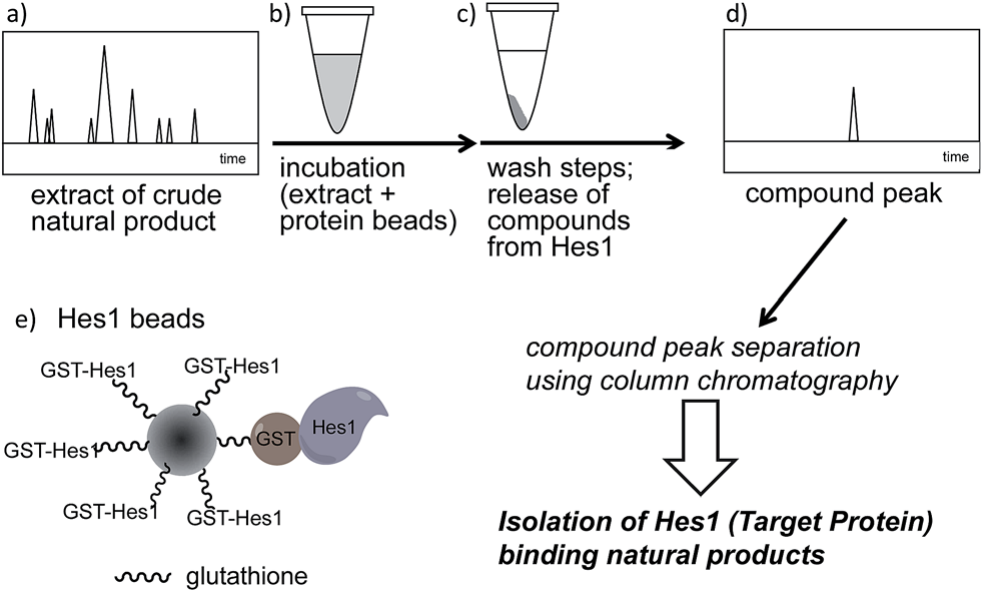
Schematic showing target protein-oriented natural product isolation by the Hes1 beads-HPLC method. (a) HPLC profile of a natural product extract; (b) incubation of the extract with Hes1 beads; (c) washing the beads and release of compounds from Hes1; (d) detection of the Hes1 binding natural product; (e) representation of the Hes1 glutathione Sepharose beads.

A schematic of an approach for Hes1-binding natural products is shown in Fig. 1. Glutathione-*S*-transferase (GST) fused Hes1-immobilized beads (GST-Hes1-beads) were mixed with our library of natural product extracts. The extracts provided many HPLC peaks, corresponding to the various natural product components (Fig. 1a). After incubation of the protein-beads with an extract, the beads were washed to remove unbound natural products. Natural product components bound to Hes1 can be released by EtOH and heating, then analyzed by HPLC (Fig. 1d). The retention times and UV absorption patterns allow the desired natural product component to be followed easily during the fractionation and isolation steps. To help choose the most promising component, compounds which bind non-specifically were identified using beads with GST immobilized at the comparable concentration as GST-Hes1 protein immobilized on the GST-Hes1 protein beads (GST-Hes1; *ca.* 3.6 nmol per bed volume 100 μl beads, GST; *ca.* 3.8 nmol per bed volume 100 μl beads). Although there have been no reports of small molecules binding to Hes1, we previously identified Hes1 dimer inhibitors from our natural product library using the Hes1 dimer plate assay.^40^ Of these inhibitors, the natural product lindbladione (**1**)^41^ inhibits Hes1 dimer suppression of DNA expression in cells.^40^ Optimization of incubation time, buffer, detergent and the method for releasing the bound compound from the Sepharose beads lead to the Hes1-beads-HPLC method (see data in **1** in ESI†).

**Fig. 2:**
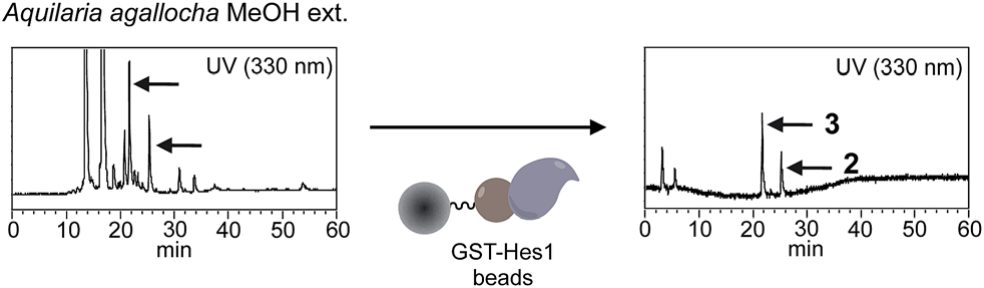
HPLC chromatograms of *A. agallocha* MeOH extract (left) and Hes1 binding natural products after screening (right). For screening, the amount of proteins on beads were controlled; GST-Hes1 beads (GST-Hes1: *ca.* 3.6 nmol), GST-beads (GST: *ca.* 3.8 nmol). The mixture of beads (bed volume 100 μl) and extract (125 μg in EtOH, 25 μl) was incubated at 4 °C for 2 h. After washing, the binding natural products were dissociated from proteins by addition of 70% EtOH and heating (100 °C, 3 min).

**Fig. 3:**
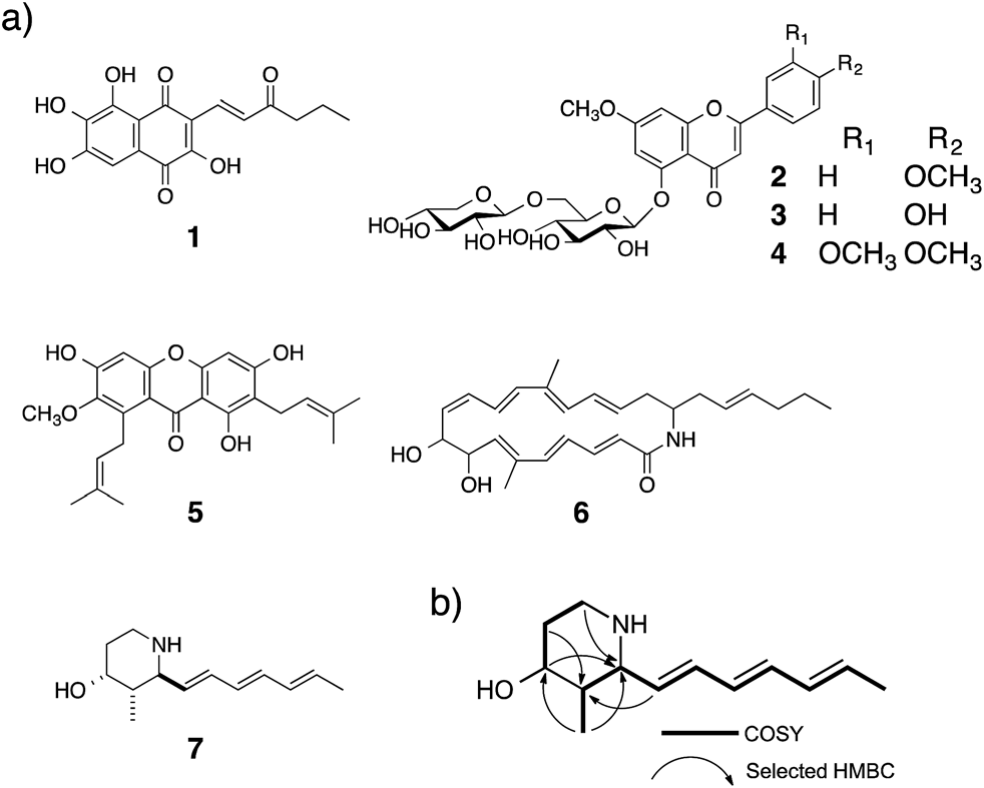
(a) Structures of isolated natural products **1–7**. (b) Key ^1^H–^1^H–COSY and HMBC data for **7**.

Using the Hes1-beads assay method, 177 extracts of tropical plants and 320 extracts of actinomycete strains were screened. The results from *Aquilaria agallocha* (leaves) collected in Thailand are shown in Fig. 2; other HPLC results of hit extracts are provided in ESI.† The left-hand HPLC profile shows the MeOH extract of *A. agallocha*; many UV-absorbing peaks are evident. After mixing with GST-Hes1 beads, followed by washing and release of the bound compounds by addition of EtOH and heating, the supernatant provided several peaks. Comparison of the HPLC results with those from GST-beads (control) led to the isolation and identification of 7,4′-di-*O*-methylapigenin 5-*O*-β-d-xylosyl-β-d-glucoside (**2**),^42^,^43^ genkwanin 5-*O*-β-d-xylosyl-β-d-glucoside (**3**),^44^ and lethedioside A (**4**)^43^ as Hes1 binders (Fig. 3a). α-Mangostine (**5**)^45^ from *Garcinia mangostana* (calyx) collected in Thailand was rapidly isolated by just one HPLC separation step. A macrolactam, BE-14106 (**6**),^46^,^47^ was isolated from an AcOEt extract of *Actinoalloteichus cyanogriseus* IFM11549 from a soil sample collected at the Sakazuki forest in Chiba, Japan. A new natural product, a hydroxypiperidine with three conjugated double bonds, was named inohanamine (**7**) and was also isolated using the HPLC peak guide approach. Compound **7** was isolated from an AcOEt extract of *Streptomyces* sp. IFM 11584 collected at Inohana Park in Chiba, Japan. The structure of **7** was determined by ^1^H- and ^13^C-NMR, HRMS, HMBC, COSY and NOE (Fig. 3b, and ESI†). The ability of the isolated compounds **2–7** to bind to Hes1 was verified using GST-Hes1 beads (see ESI†).

**Fig. 4:**
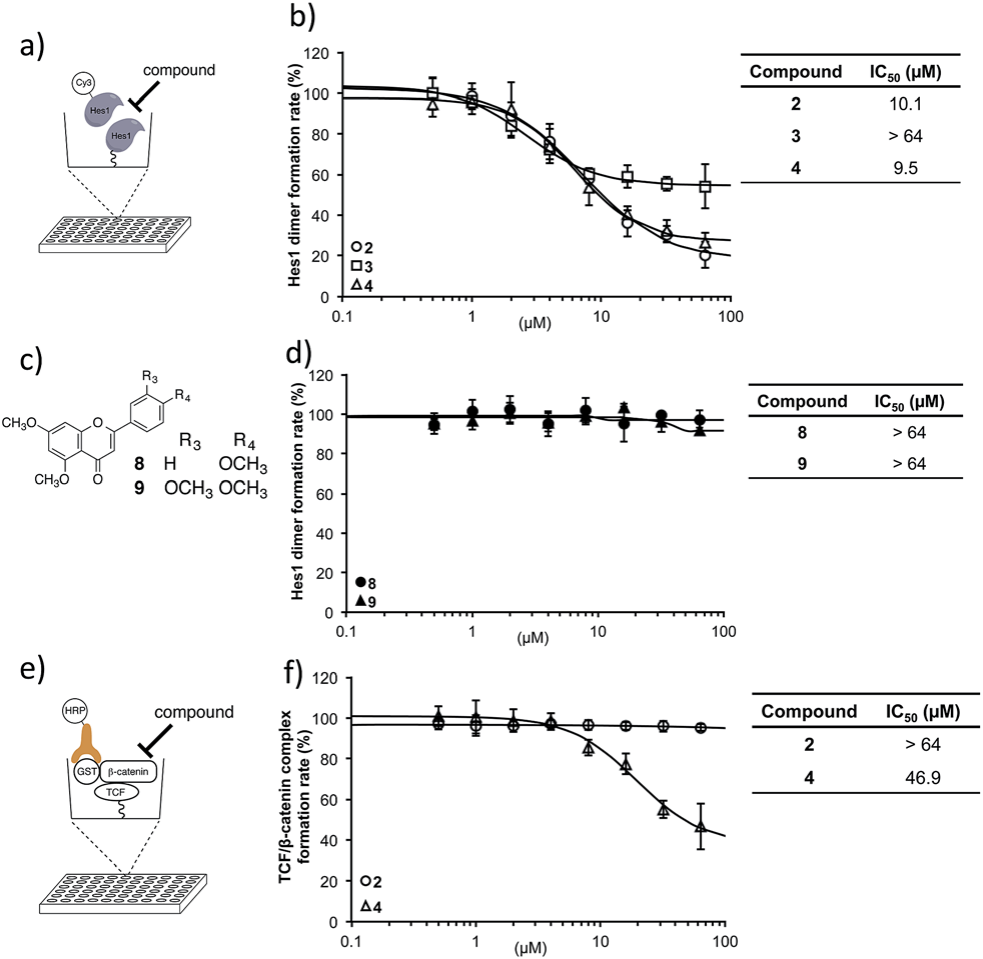
Hes1 dimer formation inhibitory activity. (a) Schematic representation of the assay for inhibitors of Hes1 dimer formation. Rat-Hes1 (residues 3-281) was exposed to Cy3-labeled Hes1. Hes1 dimer was detected through its fluorescence intensity.^40^ The assay was performed after the equilibrium of exchange between immobilized Hes1 with Cy3-Hes1 and unlabeled Hes1 which was estimated to be included as unlabeled Hes1 dimers at the immobilizing stage. (b) Hes1 dimer formation inhibition by the isolated compounds **2–4**. The background value (without Hes1, Cy3-Hes1 treated) was subtracted. (c) Structures of the flavonoid unit of active compounds **2** and **4** (**8** and **9**, respectively). (d) Flavonoid units did not inhibit Hes1 dimer formation. The background value (without Hes1, Cy3-Hes1 treated) was subtracted. (e) Elucidation of non-specific inhibition using the TCF4/β-catenin complex plate assay. Immobilized hTCF4 (residues 1-100) was exposed to GST-β-catenin (residues 128-683). The complex was detected with horseradish peroxidase (HRP) conjugated anti-GST antibody. Compounds that disrupt the TCF4/β-catenin complex reduced HRP-related chemiluminescence. (f) Compound **2** did not inhibit TCF4/β-catenin complex formation. The background value (without TCF4, antibody treated) was subtracted. Error bars show the standard deviation (*n* = 3).

With these isolated Hes1-binding natural compounds in-hand, their ability to inhibit Hes1 dimer formation was examined using the Hes1 dimer plate assay.^40^ In this technique, Hes1 was immobilized on the bottom of a microplate and Cy3-labeled Hes1 added (Fig. 4a). Hes1 dimer formation can be detected by measuring fluorescence intensity. Of the isolated Hes1 binding natural products, compounds **2** and **4** showed the highest Hes1 dimer inhibition: an IC_50_ of 10.1 and 9.5 μM, respectively (Fig. 4b). The flavones (**8**, **9**), which are the core flavanone structures of **2** and **4**, respectively (Fig. 4c), did not show Hes1 dimer inhibition, indicating that the entire structure, including the sugar, is needed for activity (Fig. 4d). Nonspecific inhibition was measured using a transcription factor, TCF (T-cell factor), and β-catenin complex, which is a key player in the transcription of Wnt signal^48^-related target genes. An ELISA (enzyme-linked immunosorbent assay) for TCF4/β-catenin complex was constructed using a slight modification of a reported method (Fig. 4e).^49^ The reliability of the assay was confirmed using a known TCF4/β-catenin complex inhibitor, calphostin C (PKF115-584)^49^ (see ESI†). Compound **2** did not show TCF/β-catenin complex inhibition, whereas **4** showed weak inhibition (Fig. 4f). This result indicated that Hes1 dimer inhibition by **2** is not due to nonspecific binding to the protein complex. Having confirmed Hes1 dimer inhibition by compound **2** (named agalloside; *Aquilaria **agallo**cha*), **2** was investigated further. The electrostatic charge distributions of compounds with similar structure (**2–4**) were examined using DFT calculations (see ESI†). Only agalloside (**2**) has an obvious electron deficient area which might be one of the reasons its activity against Hes1.

**Fig. 5:**
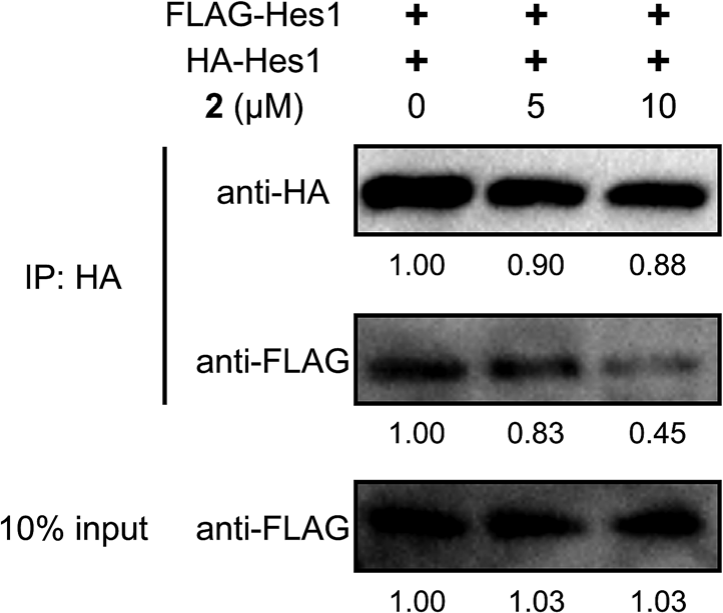
Inhibition of Hes1 dimer formation in C3H10T1/2 cells by **2** (5 and 10 μM). HA and Flag tagged Hes1 were expressed in the cells. Immunoprecipitation assays were performed using HA antibody beads. HA and Flag tagged Hes1 were recognized by each antibody, thus detecting the Hes1 dimer.

To examine Hes1 dimer inhibition in cells, HA- and Flag-tagged Hes1 expression vectors (pCI-HA-Hes1, pCI-FLAG-Hes1) were prepared and transfected into C3H10T1/2 cells (Fig. 5), according to a reported method which can detect protein homo-dimers.^50^ Immunoprecipitation (IP) assays were performed using HA antibody beads. HA- and Flag-tagged Hes1 were detected by each antibody. Treating the cells with agalloside (**2**) (5 and 10 μM) clearly decreased Flag-Hes1 dose dependently, indicating that **2** inhibits Hes1 dimer formation in cells.

### Evaluation of effects of agalloside (**2**) on NSCs

**Fig. 6:**
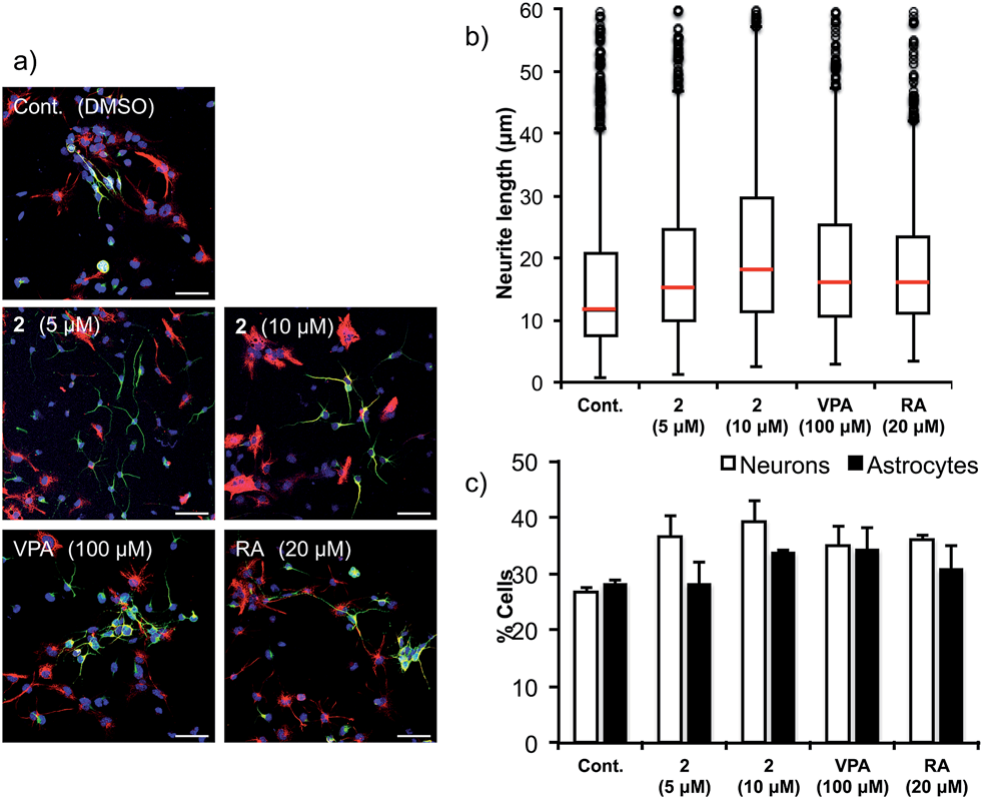
NSC differentiation-promoting activity. (a) MEB5 cells were treated with DMSO (negative control), agalloside (**2**) (5 and 10 μM), VPA (positive control; 100 μM) or RA (positive control; 20 μM) for four days. The cells were then immunostained with Tuj1 (green) for neurons, GFAP (red) for astrocytes, and TO-PRO-3 (blue) for nuclei. Scale bar: 50 μm. DMSO = dimethyl sulfoxide, VPA = valproic acid, RA = retinoic acid. VPA and RA were positive controls. (b) Effects on the length of neurites. The lengths of the neurites are shown as box plots: middle bars (red) show the median values, each box and bar shows 25% of total number of neurites. Over 1200 neurites in each sample were measured. (c) Effects on the number of neurons and astrocytes. Over 3000 cells in each sample were counted.

Next, the ability of **2** to accelerate NSCs was evaluated (Fig. 6). Multipotent mouse neural stem cells (MEB5)^51^ were treated with DMSO (control), valproic acid (100 μM), retinoic acid (20 μM) (positive controls) or agalloside (**2**) (5 and 10 μM) for four days. A confocal microscope was used to obtain images of differentiated neural cells after immunostaining class III β-tubulin (Tuj1) in neurons, and glial fibrillary acidic protein (GFAP) in astrocytes and nuclei (TO-PRO-3). The number of neurons and the length of the neurites were calculated for over 3000 cells and 800 neurites in each sample. Agalloside (**2**) exhibits potent neurite outgrowth promoting activity (Fig. 6b). The median value of neurite length (red bar) increased at a lower concentration of **2** compared to the concentration required for comparable increases by the positive controls (valproic acid and retinoic acid). Comparison of the number of neurons and astrocytes *vs.* the total number of cells showed increased neuronal differentiation with agalloside (**2**) (Fig. 6c). The differentiated neurons with **2** were 36.5% (5 μM) and 39.3% (10 μM), which represent a 36.2 and 46.6% increase compared to those of control. The differentiated astrocytes with **2** were 28.0% (5 μM) and 33.5% (10 μM), which show 0.0 and 19.6% increase compared to those of control. Agalloside (**2**) showed neuron specificity in differentiation.

**Fig. 7:**
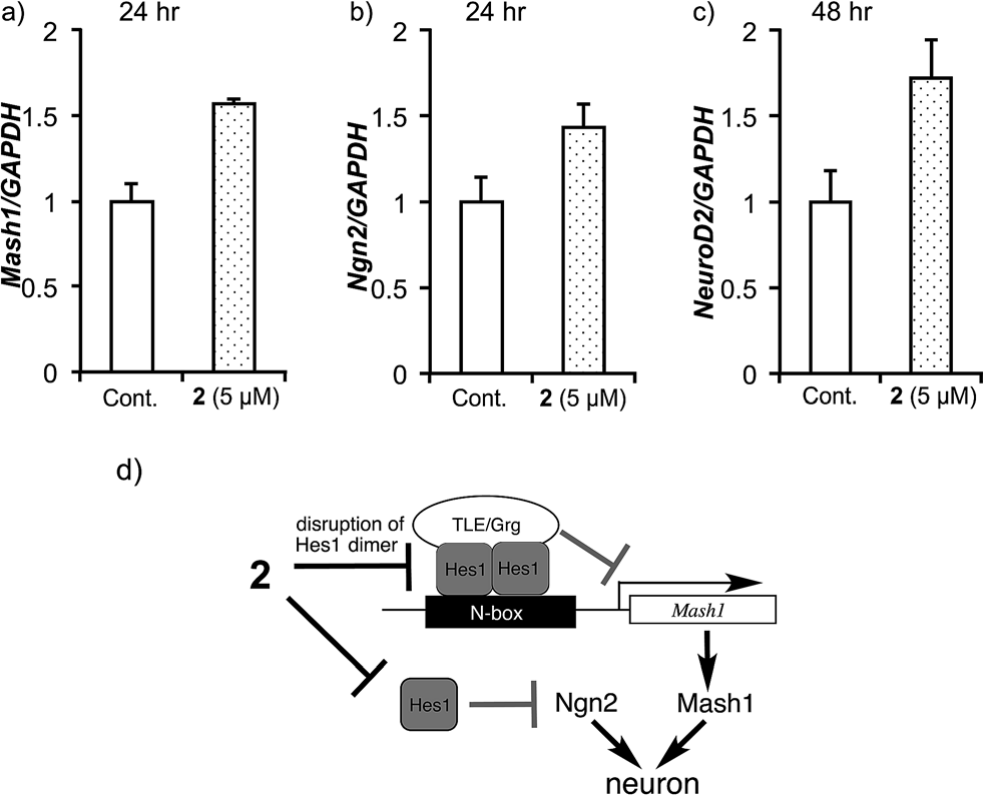
The effects of compound **2** on mRNA expression of pro-neural bHLH transcriptional activators (Mash1, Ngn2 and NeuroD2) in MEB5 cells after incubation with **2**. The mRNA levels were analysed by real-time RT-PCR (normalized to the level of GAPDH). Error bars represent the standard deviation (*N* = 3). Assays were performed in triplicate. (a) *Mash1* (after 24 h), (b) *Ngn2* (after 24 h) and (c) *NeuroD2* (after 48 h). (d) Possible mechanism of NSC differentiation promoting activity of compound **2**.

To elucidate the effect of **2** on mRNA expression of pro-neural genes (activator-type bHLH factors), the expression levels of *Mash1*, *Ngn2* and *NeuroD2* were examined (Fig. 7). It was previously reported that Hes1 represses *Mash1* expression by directly binding to the *Mash1* promoter.^52^–^54^ Real-time RT-PCR showed that agalloside (**2**) up-regulates *Mash1* after 24 h (Fig. 7a). Moreover, agalloside (**2**) cancelled the N-box dependent repression activity of Hes1 dimers^55^ in a dose-dependent manner (see ESI†). Taken together, it appears that agalloside (**2**) disrupts Hes1 dimer formation in NSCs, leading to upregulation of *Mash1*. This is the first report of the upregulation of a pro-neural gene by a Hes1 dimer inhibitor. Although it remains unknown whether the Hes1 dimer regulates directly or indirectly, Hes1 also regulates the expression of *Ngn2*.^56^ Sustained upregulation of *Ngn2* is required for neural differentiation. Increased *Ngn2* expression was clearly detected after treatment with **2** for 24 h (Fig. 7b), suggesting that agalloside (**2**) might inhibit the suppression of *Ngn2* expression by Hes1. Moreover, the neuronal specificity of **2** for the differentiation of NSCs is supported by reports that *Mash1* and *Ngn2* suppress astrocytic gene expression.^24^,^57^ *NeuroD2* is a late response activator-type bHLH factor, and *Mash1* acts upstream of *NeuroD*.^26^,^58^ Cells treated with **2** expressed *NeuroD2* more highly than the control after 48 h (Fig. 7c). The disruption of Hes1 dimer formation by **2** would increase the transcription of pro-neural genes such as *Mash1* (*Ascl1*) and *Ngn2*, which activate the transcription of neurogenesis genes such as *NeuroD2* (Fig. 7d).

### The binding region of agalloside (**2**) in Hes1

**Fig. 8:**
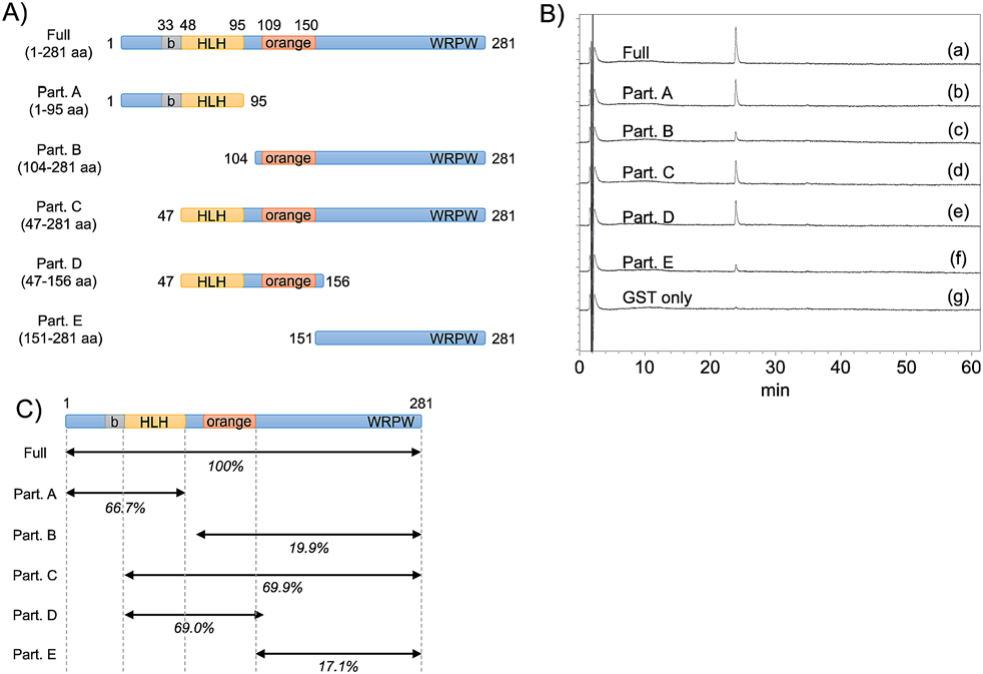
The binding ability of compound **2** to Hes1 partial proteins. All GST-Hes1 proteins were used *ca.* 3.6 nmol and GST protein was used *ca.* 3.8 nmol. (A) Schematic showing of synthesized Hes1 full and partial proteins. (B) The results of binding amount of compound **2** to each protein beads. (a) GST-Hes1 (1-281 aa) beads, (b) GST-Hes1 (1-95 aa) beads, (c) GST-Hes1 (104-281 aa) beads, (d) GST-Hes1 (47-281 aa) beads, (e) GST-Hes1 (47-156 aa) beads, (f) GST-Hes1 (151-281 aa) beads, (g) GST beads. The mass of binding compound were calculated from the area of the peaks. (C) The predicted binding ability from the results of beads assay.

To predict the binding region of agalloside (**2**) in Hes1, partial proteins of Hes1 were synthesized (Fig. 8A). Agalloside (**2**) was mixed and incubated with each protein immobilized beads (Full, 1-95aa, 104-281aa, 47-281aa, 47-156aa, 151-281aa and GST only). After washing the beads, binding compound (**2**) was released by addition EtOH. Using HPLC, the binding amounts were compared as their UV absorption (Fig. 8B). The beads with Full Hes1, Part A (1-95aa), Part C (47-281aa), Part D (47-156aa) showed significant agalloside (**2**) binding. These partial Hes1 proteins have an HLH domain. On the other hand, the amount of agalloside (**2**) decreased after mixing with the beads of Part B (104-281aa) and Part E (151-281aa), which do not have an HLH domain. Fig. 8C shows predicted binding ability from the results of beads assay. These results indicated that agalloside (**2**) binding region in Hes1 can be predicted as the HLH domain. We also predicted the interaction between the HLH domain and agalloside (**2**) by performing *in silico* docking analysis. The sugar region of agalloside (**2**) might interact and disrupt the hydrophilic interaction between Arg46, Glu76 and Lys77, which would be important to make the helix and loop units binding. Moreover, the flavonoid core seems to bind the hydrophobic pocket, which consists with Ile50, Leu54 and Leu57 of the helix (ESI†).

## Conclusions

In conclusion, we report here the first example of a naturally occurring Hes1 dimer inhibitor, agalloside (**2**), which was isolated by the TPO-NAPI method using Hes1 immobilized beads. We demonstrated the isolation of six natural products, including one new compound, by TPO-NAPI. This method is useful for isolating natural products which bind to target proteins. In principle, the binding activity of ligands to proteins gives compounds the chance for a desired bioactivity. We thus believe that this strategy using TPO-NAPI to identify modulators bHLH factors will provide good candidates for development as regenerative drugs.

## Experimental details

### A typical screening procedure

To prepare GST-Hes1 beads, GST-Hes1 (200 μg, *ca.* 3.6 nmol) in PBS was added to pre-washed glutathione Sepharose 4B beads (bed volume 100 μL, GE Healthcare) and they were mixed at 4 °C for 1 h. The GST-Hes1 beads were washed five times by NET buffer (20 mM Tris–HCl, pH 7.5, 200 mM NaCl, 1 mM EDTA), then the beads were suspended in NET buffer (250 μL). A MeOH or EtOAc extract of natural resources (125 μg in EtOH, 25 μL) was added to above GST-Hes1 freshly prepared beads (bed volume 100 μL) and the mixture was gently mixed for 2 h at 4 °C. The beads were then washed by a rotated-mixer at 4 °C for 10 min three times with NET-N buffer (NET buffer containing 0.05% Nonidet P-40, 500 μL). 70% EtOH (150 μL) was then added to the washed beads and the suspension was heated at 100 °C for 3 min. The beads were gathered by centrifugation (2000 rpm, 4 °C, 1 min) and the supernatant was centrifuged at 15 000 rpm for 15 min. The one-third of the supernatant was analyzed by HPLC. The control GST-beads were also prepared in the same procedure of GST-Hes1 beads. GST (100 μg, *ca.* 3.8 nmol) in PBS was added to pre-washed glutathione Sepharose 4B beads (bed volume 100 μL, GE Healthcare). If there is the obvious difference in the peak intensity between the results of GST-Hes1-beads and GST-beads (control), such extracts were obtained as “hit” extracts which had the Hes1 binding natural products.

### Fluorescence plate assay for inhibitors of Hes1 dimer formation

Nunc Immobilizer™ Amino 96 well plate, white (Nalge Nunc Int.) was used for immobilizing of Hes1. The wells were incubated with 100 μL of Hes1 (10 μg mL^–1^ in PBS) for 2 h at 4 °C. After the removal of protein solution, to block remaining activated units on the well, the wells were incubated for 2 h at 4 °C with 100 μL of 10 mM ethanolamine (in 100 mM Na_2_CO_3_ buffer, pH 9.6), then washed twice with 200 μL of PBST (PBS containing 0.05% Tween 20). The Hes1 bound microplate wells were incubated with 50 μL of Cy3-labeled-Hes1 in NET-N buffer (*ca.* 7 mg L^–1^, dye/protein = 0.65) for 24 h at 4 °C. After removal of protein solution, each well was washed twice with 200 μL of PBST, then each compound solution (NET-N buffer, 50 μL) was added. After incubation for 1 h at RT in the dark, each well was washed twice with 200 μL of PBST, then dried under reduced pressure 1 h in the dark. The fluorescence intensity was measured in a microplate reader (Fluoroskan Ascent, Thermo). The Cy3 dye was excited at 530 nm and emitted at 590 nm. Usually, the assays were carried out in three individual wells, and the mean value and SD were calculated.

## Supplementary Material

Supplementary information
