# Supplementary material for: Hes1 inhibitor isolated by target protein oriented natural products isolation (TPO-NAPI) of differentiation activators of neural stem cells
Source: Chem Sci. 2015 Dec 1;7(2):1514–20. doi: 10.1039/c5sc03540f (PMC5963671; doi:10.1039/c5sc03540f)
Supplement: Supplementary file 1 [file SC-007-C5SC03540F-s001.pdf]

Electronic Supplementary Material (ESI) for Chemical Science.
This journal is © The Royal Society of Chemistry 2015

Hes1 Dimer Inhibitor Isolated by Target Protein Oriented Natural Products Isolation (TPO-NAPI) for Differentiation Activators of Neural Stem Cells

Midori A. Arai*, Naoki Ishikawa, Mitsuha Tanaka, Kenji Uemura, Noriko Sugimitsu, Akiko Suganami, Yutaka Tamura, Takashi Koyano, Thaworn Kowithayakorn, Masami Ishibashi*

Supporting Information

General experiment procedures

NMR spectra were recorded on JEOL A400, ECP400, ECP600 and ECA600 spectrometers in a deuterated solvent whose chemical shift was taken as an internal standard. Electrospray ionization mass spectra (ESIMS) were obtained using JEOL JMS-T100LP and SHIMADZU LCMS-2020. Optical rotations were measured using a JASCO P-1020 polarimeter. UV spectra were measured in a SHIMADZU UV mini-1240 spectrometer. IR spectra were measured using the ATR (Attenuated Total Reflection) method in a JASCO FT-IR 230 spectrophotometer. HPLC was carried out on a Waters 600 pump that was equipped with a Waters 2996 Photodiode Array Detector or SHIMADZU LC-20AD pump that was equipped with a SHIMADZU

SPD-M10AVP Photodiode Array Detector. LC-MS analysis was carried out on a SHIMADZU LC-20AT pump that was equipped a SHIMADZU SPD-M20A Photodiode Array Detector and SHIMADZU LCMS-2020. Column chromatography was performed using silica gel PSQ100B, Chromatorex ODS, Chromatorex DIOL (Fuji Silysia Chemical Ltd., Kasugai, Japan), silica gel 60N (Kanto Chemical Co., Inc., Tokyo, Japan) and Sephadex LH-20 (GE Healthcare life sciences, Tokyo, Japan). Analytical HPLC was performed using Mightysil RP-18 GP (Kanto Chemical Co., Inc.), CAPCELL PAK C18 MGII (Shiseido Co., Ltd., Yokohama, Japan), or COSMOSIL 5C_{18}-AR-II (Nacalai tesque, Inc., Kyoto, Japan). Preparative HPLC was performed using CAPCELL PAK C18 ACR, CAPCELL PAK C18 MGII (Shiseido Co., Ltd.), YMC-Pack Pro C18 (YMC, Co., Ltd., Kyoto, Japan), or Develosil C30-UG-5 (Nomura Chemical Co., Ltd., Seto, Japan).

Plasmids

Plasmid pGEX-ratHes$1_{3\text{-}281}$ have been described previously.[1] Plasmids pCI-Hes1 and pN6-βA-luc were gift from Prof. Ryoichiro Kageyama, Kyoto University, Japan). HA or FLAG tag fused at N-terminal a rat Hes1 (full length, aa 1-281) were prepared by subcloning pCI-Hes1 plasmid as a template by PCR. The PCR-amplified DNA fragments were digested with *Nhe*I and *Eco*RI and ligated with pCI vector (Promega) digested with *Nhe*I and *Eco*RI to generate pCI-HA-Hes$1_{1\text{-}281}$ plasmid or

pCI-FLAG-Hes1$_{1\text{-}281}$ plasmid. GST fused at N-terminal a mouse β-catenin (aa 128-683) was prepared by subcloning pBSSK-β-catenin plasmid (a gift from Prof. Tetsu Akiyama, Tokyo University, Japan) as a template by PCR. The PCR-amplified DNA fragments were digested with *Bam*HI and *Sal*I and ligated with pGEX-6P-1 vector (GE Healthcare) digested with *Bam*HI and *Sal*I to generate pGEX-mβ-catenin$_{128\text{-}683}$ plasmid. GST fused at N-terminal a human TCF4E (aa 1-100) was prepared by subcloning pBSSK-hTCF4E (a gift from Prof. Tetsu Akiyama) as a template by PCR. The PCR-amplified DNA fragments were digested with *Eco*RI and *Xho*I and ligated with pGEX-6P-1 vector digested with *Eco*RI and *Xho*I to generate pGEX-hTCF4E$_{1\text{-}100}$ plasmid. GST fused at N-terminal a rat Hes1 (full length, 1-281 aa) and truncated rat Hes1 (Part. A; 1-95 aa, Part. B; 104-281 aa, Part. C; 47-281 aa, Part. D; 47-156 aa, and Part. E; 151-281 aa) were prepared by subcloning pCI-Hes1 plasmid as a template by PCR. The PCR-amplified DNA fragments were digested with *Bam*HI and *Eco*RI and ligated with pGEX-6P-1 vector digested with *Bam*HI and *Eco*RI to generate various lengths of pGEX-ratHes1 plasmids. The primers for PCR were listed in **Table S1**.

1. Arai, M. A., Masada, A., Ohtsuka, T., Kageyama, R., and Ishibashi, M. (2009) The first Hes1 dimer inhibitors from natural products, *Bioorg. Med. Chem. Lett. 19*, 5778-5781.

Table S1. Oligonucleotide primers used for subcloning in this work. The underlined nucleotides are restriction sites.

Plasmids	Primers	Sequences	Restriction sites
pCI-HA-Hes1$_{1\text{-}281}$	HA-Hes1-F	5'-AGGCTAGCACCATGTACCCATACGATGTTCCAGATTACGCTATGCCAGCTGATATAATG-3'	*Nhe*I
	HA-Hes1-R	5'-AGGAATTCAGTTTATGATTAGCAGTG-3'	*Eco*RI
pCI-FLAG-Hes1$_{1\text{-}281}$	FLAG-Hes1-F	5'-AGGCTAGCACCATGGACTACAAGGATGACGATGACAAGATGCCAGCTGATATAATG-3'	*Nhe*I
	FLAG-Hes1-R	5'-AGGAATTCAGTTTATGATTAGCAGTG-3'	*Eco*RI
pGEX-mβ-catenin$_{128\text{-}683}$	β-catenin-F	5'-AGGGATCCCCATCACAGATGTTG-3'	*Bam*HI
	β-catenin-R	5'-AGGTCGACTCAGAAGAGGGAACT-3'	*Sal*I
pGEX-hTCF4E$_{1\text{-}100}$	TCF4E-F	5'-AGGAATTCATGCCGCAGCTGAAC-3'	*Eco*RI
	TCF4E-R	5'-AGCTCGAGTCAATACGGTGGCCC-3'	*Xho*I
pGEX-Hes1$_{1\text{-}281}$ (Full)	Full-F	5'-AGGGATCCATGCCAGCTGATATAATG-3'	*Bam*HI
	Full-R	5'-AGGAATTCTCAGTTCCGCCACGGCCT-3'	*Eco*RI
pGEX-Hes1$_{1\text{-}95}$ (Part. A)	Part. A-F	5'-AGGGATCCATGCCAGCTGATATAATG-3'	*Bam*HI
	Part. A-R	5'-AGGAATTCTCACTGCGCCCGCTGCAGGTT-3'	*Eco*RI
pGEX-Hes1$_{104\text{-}281}$ (Part. B)	Part. B-F	5'-AGGGATCCCCGAGCGTGTTGGGGAAG-3'	*Bam*HI
	Part. B-R	5'-AGGGATCCATGCCAGCTGATATAATG-3'	*Eco*RI
pGEX-Hes1$_{47\text{-}281}$ (Part. C)	Part. C-F	5'-AGGGATCCCGGGCAAGAATAAATGAA-3'	*Bam*HI
	Part. C-R	5'-AGGGATCCATGCCAGCTGATATAATG-3'	*Eco*RI
pGEX-Hes1$_{47\text{-}156}$ (Part. D)	Part. D-F	5'-AGGGATCCCGGGCAAGAATAAATGAA-3'	*Bam*HI
	Part. D-R	5'-AGGAATTCTCAGGGGTAGGTCATGGCGTT-3'	*Eco*RI
pGEX-Hes1$_{151\text{-}281}$ (Part. E)	Part. E-F	5'-AGGGATCCAACGCCATGACCTACCCC-3'	*Bam*HI
	Part. E-R	5'-AGGGATCCATGCCAGCTGATATAATG-3'	*Eco*RI

Expression and purification of recombinant GST fused and cleaved proteins

E. coli strain JM109 (Nippon Gene Co., Ltd., Tokyo, Japan) serves as hosts for pGEX-Hes1$_{3\text{-}281}$, pGEX-6P-1, pGEX-mβ-catenin$_{128\text{-}683}$ and pGEX-hTCF4E$_{1\text{-}100}$. An overnight plateau phase culture of JM109 was inoculated to fresh LB medium (Invitrogen) containing 100 mg L^{-1} ampicillin. Cells were grown at 37ºC to a density of 0.6 (OD_{600}) and induced of protein synthesis by addition of 0.1 mM IPTG (isopropyl-1-thio-*β*-D-galactopyranoside) followed by incubation for an additional 4-8 h at 18ºC. The cells were harvested by centrifugation and lysed by sonication. After incubation with 1% Triton X-100 for 30 min at 4ºC, the lysate was centrifuged at 4000

rpm for 10 min at 4ºC. The resulting supernatant was added to pre-washed glutathione sepharose 4B and gently mixed for 1 h at 4ºC. The beads obtained after centrifugation (2000 rpm, 5 min, at 4ºC) were washed with PBS or WE buffer (20 mM Tris-HCl, pH 7.5, 2 mM $MgCl_2$ and 0.1 mM DTT). The recombinant proteins were eluted with 50 mM glutathione buffer (50 mM Tris-HCl, pH 8.8) and then dialyzed against PBS or NET buffer using Slide-A-Lyzer® Dialysis Cassette (Thermo). The GST was cleaved from recombinant protein by PreScission protease (GE Healthcare) for 4 h at 4ºC in cleavage buffer (50 mM Tris-HCl, pH 7.5, 150 mM NaCl, 1 mM EDTA and 1 mM DTT), then dialyzed against PBS buffer. The protein concentration was determined by Micro BCA Protein Assay Kit (Thermo). The recombinant proteins were estimated to be greater than 90% pure by SDS-PAGE.

A typical screening procedure

To prepare GST-Hes1 beads, GST-Hes1 (200 μg, ca. 3.6 nmol) in PBS was added to pre-washed glutathione sepharose 4B beads (bed volume 100 μL, GE Healthcare) and they were mixed at 4ºC for 1 h. The GST-Hes1 beads were washed five times by NET buffer (20 mM Tris-HCl, pH 7.5, 200 mM NaCl, 1 mM EDTA), then the beads were suspended in NET buffer (250 μL). A MeOH or EtOAc extract of natural resources (125 μg in EtOH, 25 μL) was added to above GST-Hes1 freshly prepared beads (bed volume 100 μL) and the mixture was gently mixed for 2 h at 4 ºC. The beads were then

washed by a rotated-mixer at 4ºC for 10 min three times with NET-N buffer (NET buffer containing 0.05% Nonidet P-40, 500 μL). 70% EtOH (150 μL) was then added to the washed beads and the suspension was heating at 100ºC for 3 min by heating block. The beads were gathered by centrifugation (2000 rpm, 4ºC, 1 min) and the supernatant was centrifuged at 15000 rpm for 15 min. The one-third of the supernatant was analyzed by HPLC.

The control GST-beads were also prepared in same procedure of GST-Hes1 beads. GST (100 μg, ca. 3.8 nmol) in PBS was added to pre-washed glutathione sepharose 4B beads (bed volume 100 μL, GE Healthcare).

This method was constructed by lindobladione as a positive control (Figure S1).

If there is the obvious difference in the peak intensity between the results of GST-Hes1-beads and GST-beads (control), such extracts were obtained as "hit" extracts which had the Hes1 binding natural products. HPLC charts of hit extracts (Figure S2).

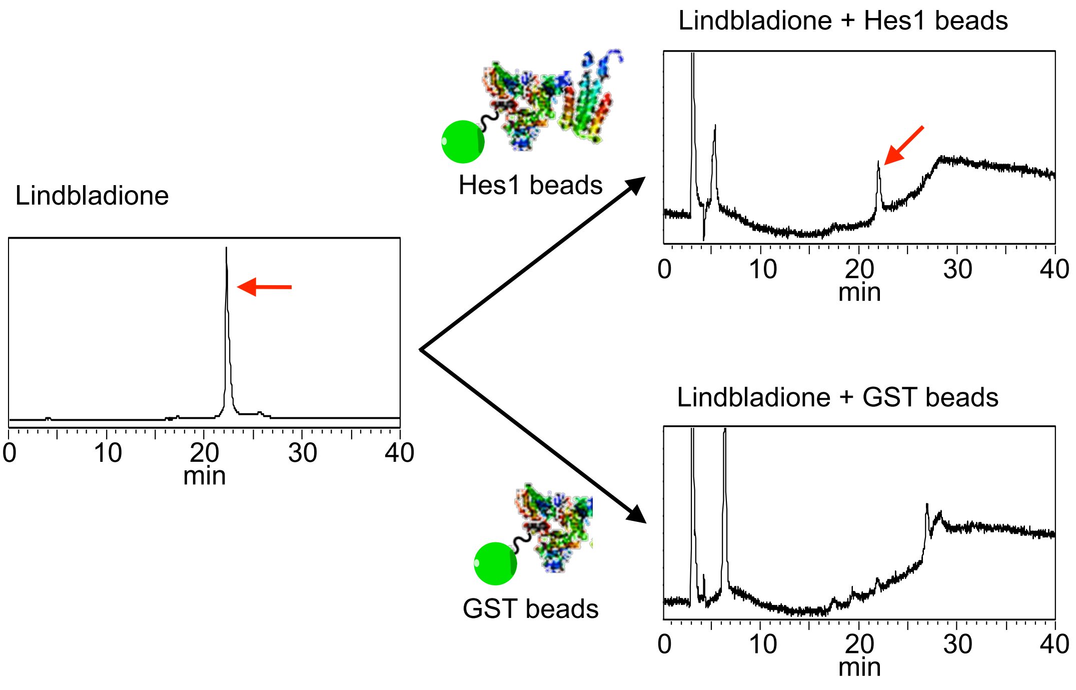

Figure S1. Hes1-beads assay by lindbladione (a positive control). Lindbladione (12.5 nmol in DMSO) was mixed and incubated with GST-Hes1 beads (ca. 3.6 nmol) and GST beads (ca. 3.8 nmol).

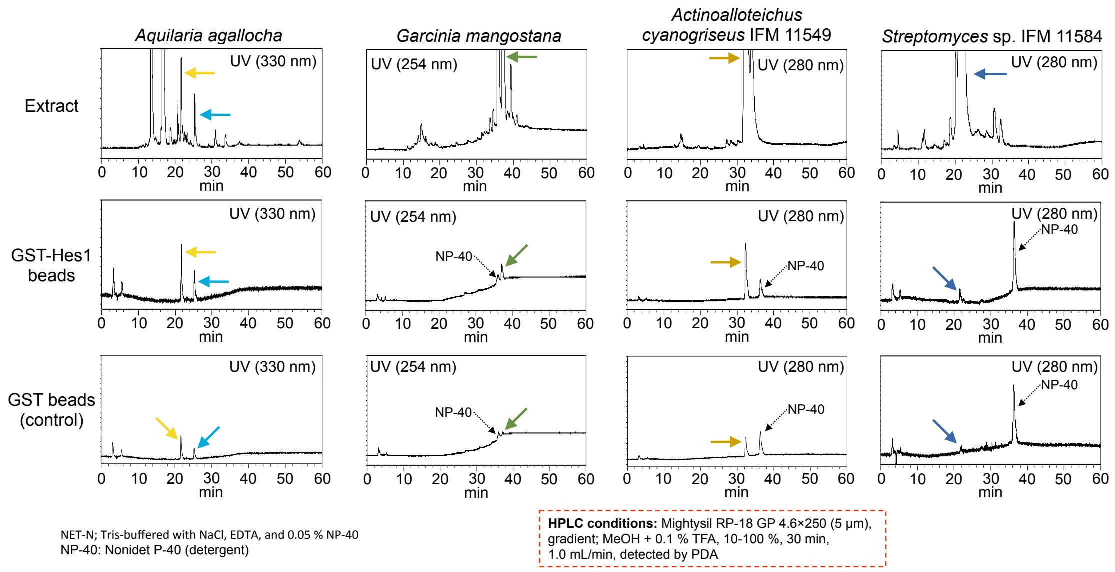

Figure S2. The HPLC results of screening. GST-Hes1 beads (ca. 3.6 nmol) and GST beads (ca. 3.8 nmol).

Plant materials

Leaves of *Aquilaria agallocha* and calyx of *Garcinia mangostana* were collected in Thailand. Voucher specimens (KKP279 for *A. agallocha* and KKP173 for *G. mangostana*) were deposited at the Department of Natural Products Chemistry, Graduate School of Pharmaceutical Sciences, Chiba University.

Microbial strain

Actinoalloteichus cyanogriseus IFM 11549 and *Streptomyces* sp. IFM 11584 were separated from soil samples collected at the Sakazuki forest and at the Inohana park, Chiba city, Japan. The identification was carried out by Professor Tohru Gonoi (Medical Mycology Research Center, Chiba University, Japan), where a voucher specimens were deposited with code IFM 11549 and IFM 11584, respectively.

Extraction and isolation from plant materials.

The air-dried leaves of *A. agallocha* (32.5 g) were extracted with MeOH overnight at RT. The MeOH extract of *A. agallocha* (8.4 g) was subjected to Diaion HP-20 column chromatography (55 × 300 mm) and eluted with gradient mixtures of MeOH-acetone (1/0-0/1) to give two fractions (frs. 1A-1B) (**Figure S3a**). Fraction 1A (6.1 g) was suspended in H_2O-MeOH (90/10, 300 mL) and partitioned with hexane (300 mL × 3), EtOAc (300 mL × 3) and *n*BuOH (300 mL × 3). The *n*BuOH-soluble fraction (1.8 g)

was subjected to ODS flash column chromatography (50 × 230 mm) and eluted with gradient mixtures of H_2O-MeOH (3/2-0/1) to give eight fractions (frs. 2A-2H). Fr. 2G (16.4 mg) was purified by reverse-phase HPLC (CAPCELL PAK C18 ACR 5 µm, 6.0 × 250 mm; eluent, H_2O-MeOH (1/1); flow rate, 1.5 mL/min; detection, UV at 330 nm) to afford compound **2** (9.4 mg, t_R 25.3 min). A part of fr. 2E (26.8 mg) was subjected to reverse-phase HPLC (YMC-Pack Pro C18, 10 × 250 mm; eluent, H_2O-MeOH (1/1); flow rate, 2.0 mL/min; detection, UV at 330 nm) to give 5 fractions (frs. 4A-4E). Compound **3** (2.7 mg, t_R 22.0 min) was obtained by recrystallizing from fr. 4B (8.2 mg) from H_2O-MeOH (1/9). Fr. 2F (42.7 mg) was separated by reverse-phase HPLC (Develosil C30-UG-5, 10 × 250 mm; eluent, H_2O-MeOH (35/65); flow rate, 2.0 mL/min; detection, UV at 330 nm) to afford compound **2** (6.5 mg, t_R 30.0 min), compound **3** (4.0 mg, t_R 15.6 min) and compound **4** (7.8 mg, t_R 22.9 min).

The Hes1 binding ability was re-checked with isolated pure natural products with GST-Hes1 beads by the procedure of screening (**Figure S3b**).

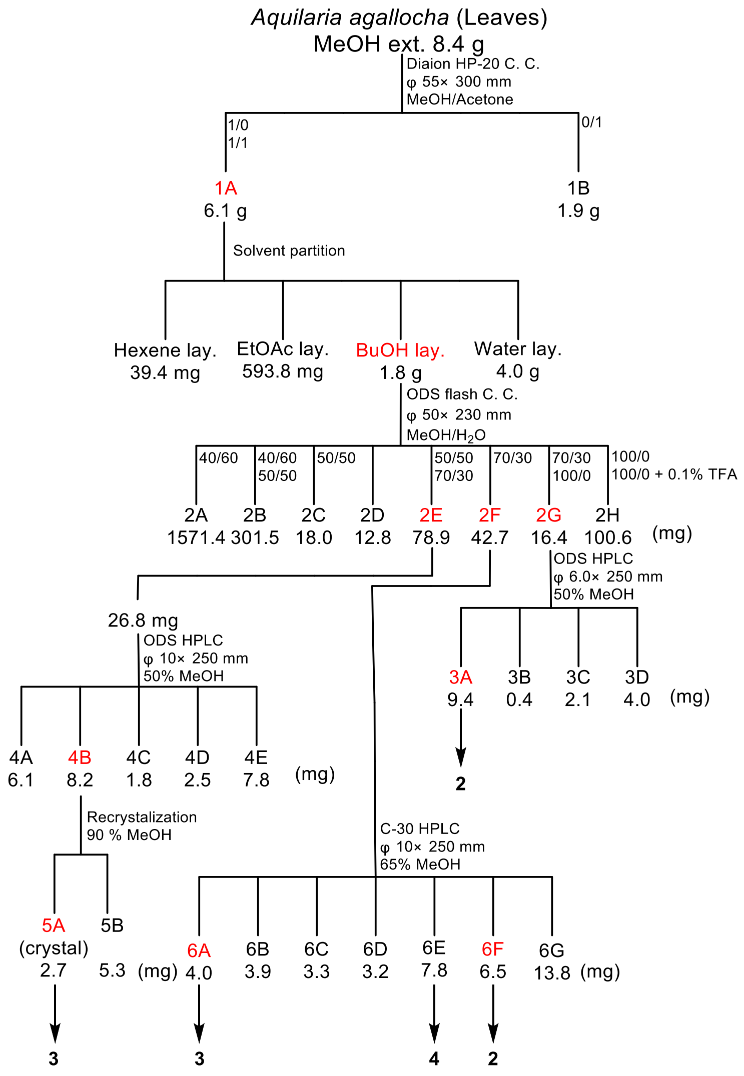

Figure S3a. Isolation scheme of *A. agallocha.* Red shows the fractions including the target peaks. Red; target peak was included.

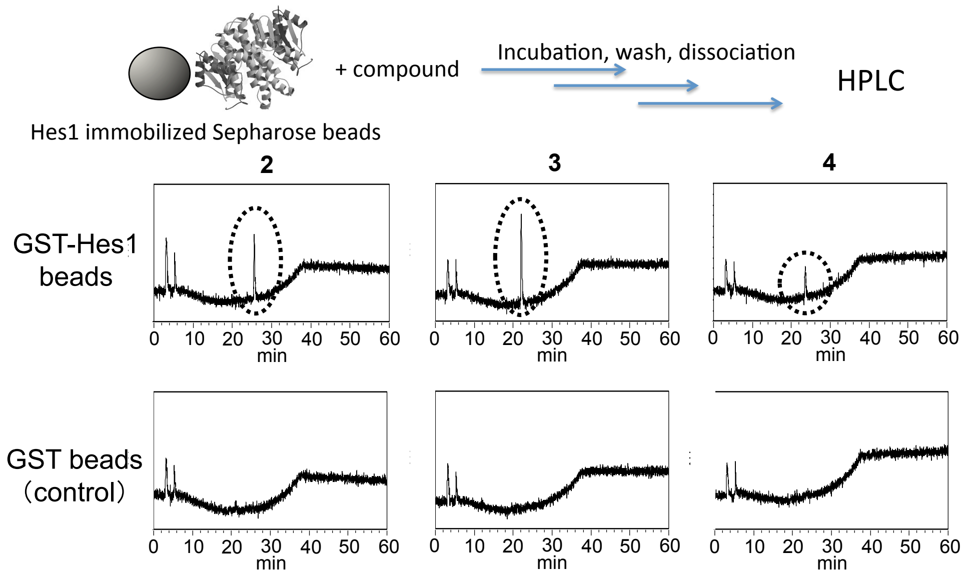

Figure S3b. Re-check binding ability to Hes1 of **2-4**. Compounds **2**-**4** (12.5 nmol in DMSO) were mixed and incubated with GST-Hes1 beads (ca. 3.6 nmol) and GST beads (ca. 3.8 nmol) respectively.

The air-dried calyx of *G. mangostana* (33.1 g) was extracted with MeOH overnight at RT (**Figure S4a**). A part of MeOH extract of *G. mangostana* (11.5 mg) was separated by reverse-phase HPLC (CAPCELL PAK C18 MGII, 10 × 250 mm; eluent, gradient mixtures of H_2O/MeOH (1/9-0/1); flow rate, 2.0 mL/min; detection, UV at 254 nm) to afford compound **5** (1.5 mg, t_R 41.0 min).

The Hes1 binding ability was re-checked with isolated pure natural products with GST-Hes1 beads by the procedure of screening (**Figure S4b**).

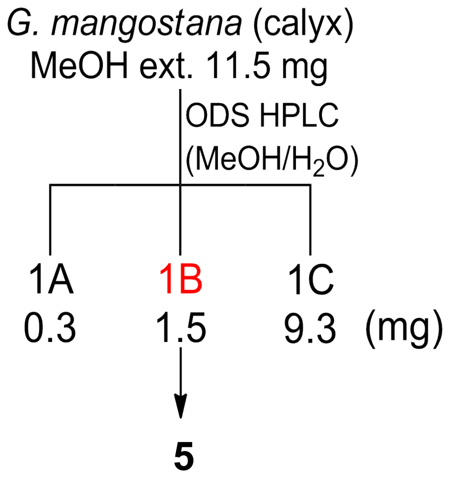

Figure S4a. Isolation scheme of *G. Mangostana*. Red; target peak was included.

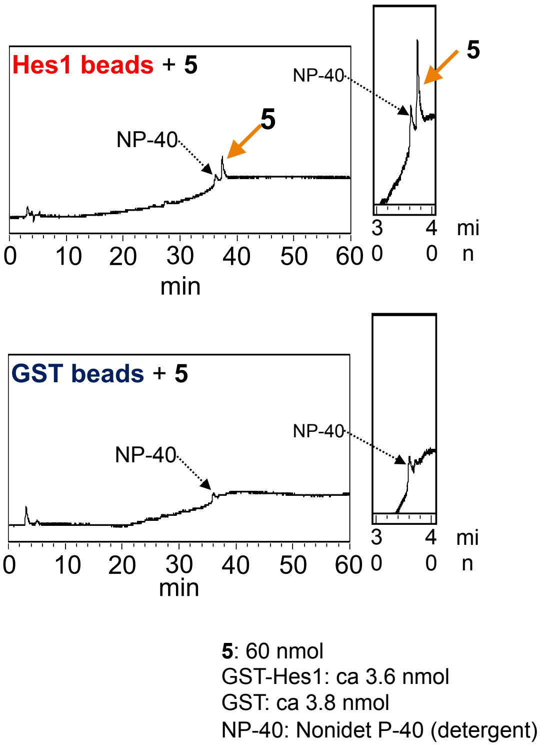

Figure S4b. Re-check binding ability to Hes1 of **5.** Compound **5** (60 nmol in DMSO) was mixed and incubated with GST-Hes1 beads (ca. 3.6 nmol) and GST beads (ca. 3.8 nmol).

Fermentation, extraction and isolation from actinomycetes

Spores of *A. cyanogriseus* IFM 11549 growing on solid Waksman medium were inoculated into 2 × 500 mL Sakaguchi flasks each containing 100 mL of liquid Waksman medium and then incubated at 27 ºC for 5 days with reciprocal shaking at 120 rpm to produce seed culture. The seed culture (20 mL) was then inoculated into each of 6 × 3 L flasks each containing 750 mL of liquid Waksman medium, and incubated at 27 ºC for 5 days with reciprocal shaking at 120 rpm to get 4.5 L of fermentation broths. Liquid Waksman medium composed of 2% glucose, 0.5% peptone, 0.5% meat extract, 0.3% yeast extract, 0.5% NaCl, 0.3% $CaCO_3$; 1.5% agar was added for solidification. The culture broth (4.5 L) was harvested and centrifuged at 3000 rpm for 20 min to separate the mycelium and supernatant. The supernatant was concentrated in vacuo to 500 mL, and then extracted with EtOAc (500 mL × 3). The mycelium was extracted with MeOH. After removal of MeOH, the extract was suspended in H_2O-MeOH (1/9, 300 mL), and partitioned with EtOAc (300 mL × 3). The EtOAc-soluble fraction of the mycelium (190.2 mg) was subjected to silica gel flash column chromatography (25 × 310 mm) and eluted with gradient mixtures of $CHCl_3$-MeOH (1/0-0/1) to give nine fractions (frs. 1A-1I). Fr. 1D (9.9 mg) was suspended in mixture of hexane-acetone (1/1) to give two fractions (frs. 2A-2B). Fr. 2A (hexane-acetone-insoluble fraction, 2.5 mg) was separated by LH-20 column chromatography (24 × 140 mm) and eluted with

CHCl3-MeOH (1/1) to afford compound **6** (1.2 mg). The EtOAc-soluble fraction of the supernatant (780.5 mg) was subjected to silica gel column chromatography (45 × 290 mm) and eluted with gradient mixtures of $CHCl_3$-MeOH (1/0-0/1) to give seven fractions (frs. 4A-4G). Fr. 4C (121.9 mg) was suspended in mixture of hexane-acetone (1/1) to afford compound **6** (1.2 mg, precipitation).

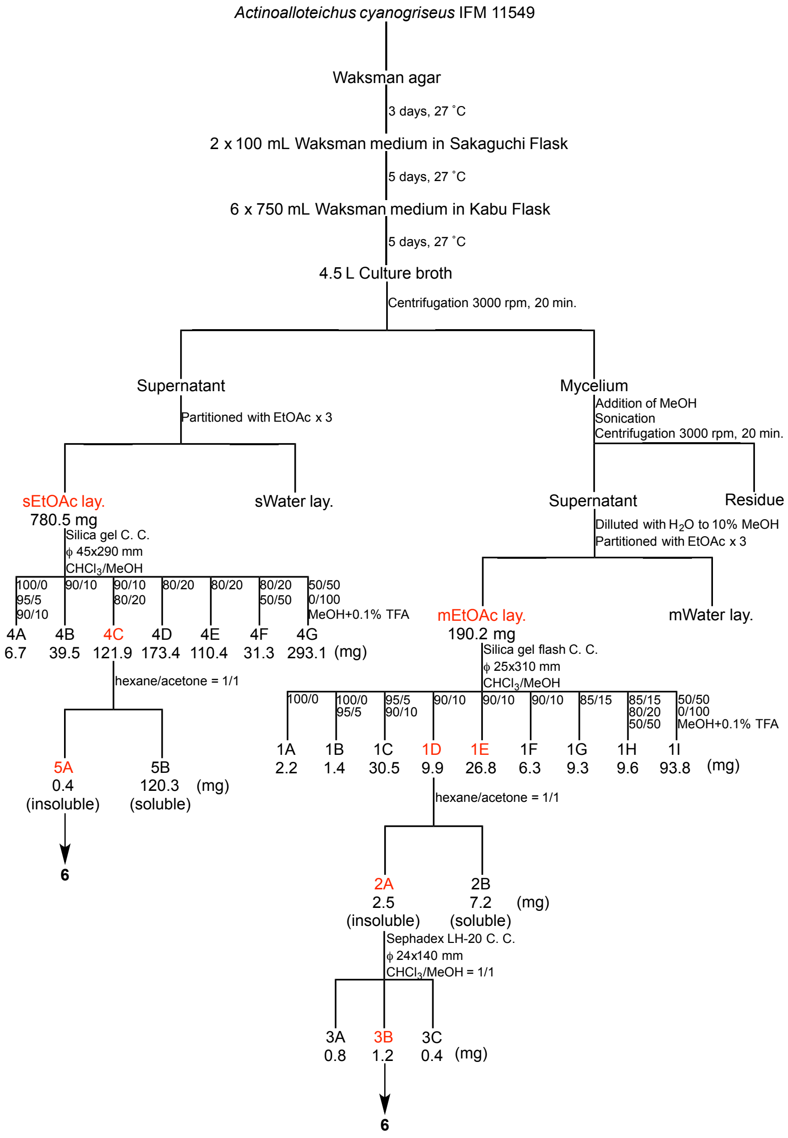

Figure S5a. Isolation scheme of *A. cyanogriseus* IFM 11549. Red; target peak was included.

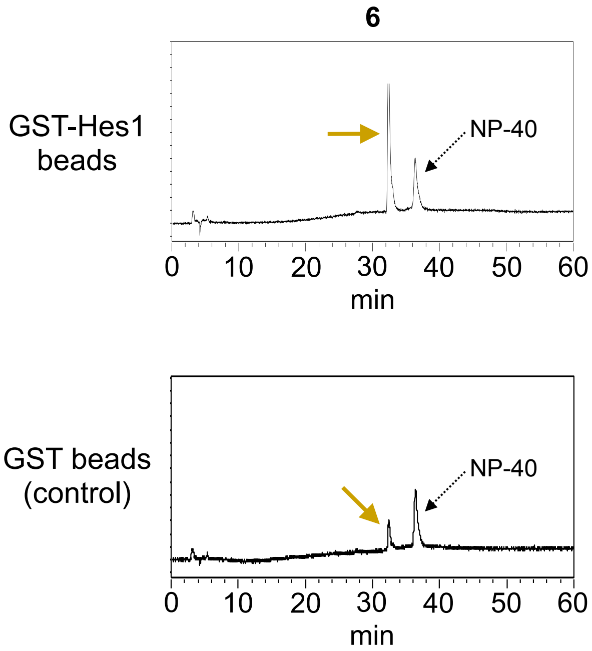

Figure S5b. Re-check binding ability to Hes1 of **6**. Compound **6** (12.5 nmol) was mixed and incubated with GST-Hes1 beads (ca. 3.6 nmol) and GST beads (ca. 3.8 nmol).

Spores of *Streptomyces* sp. IFM 11584 growing of solid Waksman medium were inoculated into 2 × 500 mL Sakaguchi flasks each containing 100 mL of liquid Waksman medium and then incubated at 27ºC for 5 days with reciprocal shaking at 120 rpm to produce seed culture. The seed culture (20 mL) was then inoculated into each of 10 × 3 L flasks each containing 750 mL of liquid Waksman medium, and incubated and 27ºC for 6 days with reciprocal shaking at 120 rpm. After the incubation, each volume of acetone was added to the culture broth and further incubated for 3 hr to kill the *Streptomyces* and to extract the active substances from the mycelia. Thereafter the broth

was filtered and concentrated in vacuo to 7.5 L. The pH value of the crude aqueous solution was adjusted to 10 by 10% Na_2CO_3 aq, and then extracted with EtOAc (7.5 L × 3). The EtOAc-soluble fraction (1.0 g) was subjected to silica gel column chromatography (42 × 306 mm) and eluted with gradient mixtures of NH_4OH saturated $CHCl_3$-MeOH (1/0-0/1) to give eleven fractions (frs. 1A-1K). A part of fr. 1C (86.6 mg) was subjected to DIOL silica gel column chromatography (26 × 300 mm) and eluted with gradient mixtures of EtOAc-MeOH (1/0-0/1) to afford compound **9** (26.1 mg).

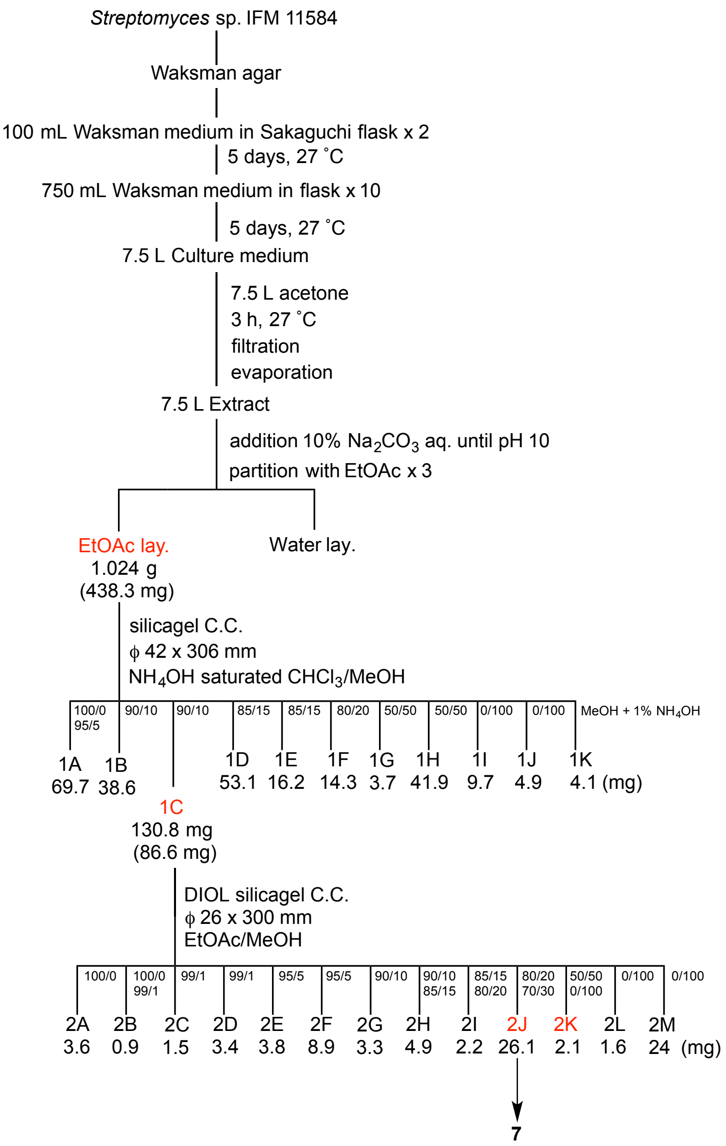

Figure S6. Isolation scheme of *Streptomyces* sp. IFM 11584. Red; target peak was included.

Preparation of Cy3-labeled Hes1 protein

Cy3-labeled Hes1 was prepared by mixing of protein in PBS with Cy3 monofunctional dye reagent (succinimidylester type; GE Healthcare) in DMSO for 30 min at 4ºC in the dark. The above dye was prepared as 1 vial of Cy3 dye reagent in DMSO (50 μL), and stored at -20ºC. Typically, 0.5 μL of DMSO solution was added to 1 mL of protein solution (10 mg L^{-1}) in PBS. After incubation, the mixture was directly dialyzed with

NET buffer at 4ºC to remove excess unreacted dye reagent. After 3 h dialysis, NET buffer was replaced with fresh NET buffer and dialyzed overnight. The ratio of dye/protein was calculated from the absorption for Cy3 (ε 150000 M^{-1} cm^{-1} at 552 nm). Because it was revealed that 30% reduction of protein concentration after dialysis, concentration of Cy3-Hes1 protein estimated ca. 7 mg L^{-1}.

Fluorescence plate assay for inhibitors of Hes1 dimer formation

Nunc Immobilizer^{TM} Amino 96 well plate, white (Nalge Nunc Int.) was used for immobilizing of Hes1. The wells were incubated with 100 μL of Hes1 (10 mg L^{-1} in PBS) for 2 h at 4ºC. After the removal of protein solution, to block remaining activated unit on the well, the wells were incubated for 2 h at 4ºC with 100 μL of 10 mM ethanolamine (in 100 mM Na_2CO_3 buffer, pH 9.6), then washed twice with 200 μL of PBST (PBS containing 0.05% Tween 20). The Hes1 bound microplate wells were incubated with 50 μL of Cy3-labeled-Hes1 in NET-N buffer (ca. 7 mg L^{-1}, dye/protein = 0.65) for 24 h at 4ºC (see Fig. S7). After removal of protein solution, each well was washed twice with 200 μL of PBST, then each compound solution (NET-N buffer, 50 μL) was added. After incubation for 1 h at RT in the dark, each well was washed twice with 200 μL of PBST, then dried under reduced pressure 1 h in the dark. The fluorescence intensity was measured in a microplate reader (Fluoroskan Ascent, Thermo). The Cy3 dye was excited at 530 nm and emitted at 590 nm. Usually, the

assays were carried out in three individual wells, and the mean value and SD were calculated.

The assay was performed after the equilibrium of exchange between the dimer of immobilized Hes1 with Cy3-Hes1 and the dimer of immobilized Hes1 with unlabeled Hes1 which was estimated to be included at the immobilizing stage. To determine the appropriate incubation time with Cy3-Hes1, the incubate time was examined (Figure S7). The exchange between excess Cy3-Hes1 and unlabeled Hes1 was accomplished after 24 h. Most of dimers were estimated as the dimer of immobilized Hes1 with Cy3-Hes1, because the amount of Cy3-Hes1 is huge excess amount against immobilized Hes1.

This assay was constructed by lindobladione as a positive control (Figure S7).

The results of Hes1 dimer inhibition were shown (Figure S8a,b).

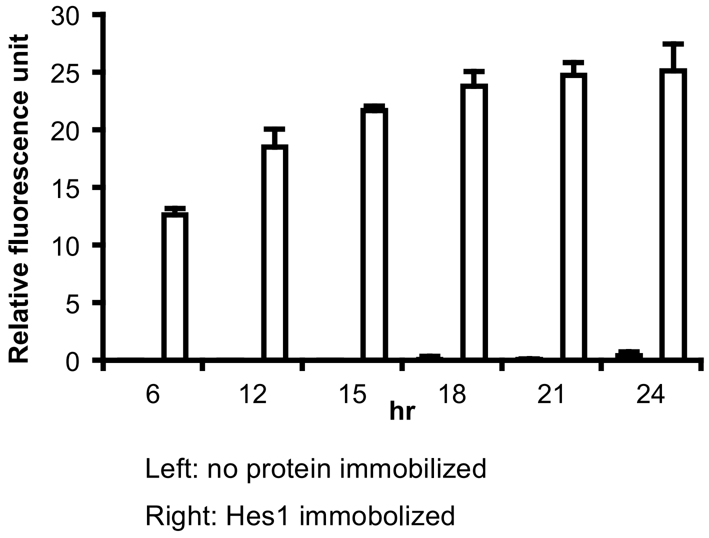

Figure S7. Examination of incubation time with Cy3-Hes1 and immobilized Hes1. The exchange between Cy3-Hes1 and unlabeled Hes1 was accomplished after 24 h.

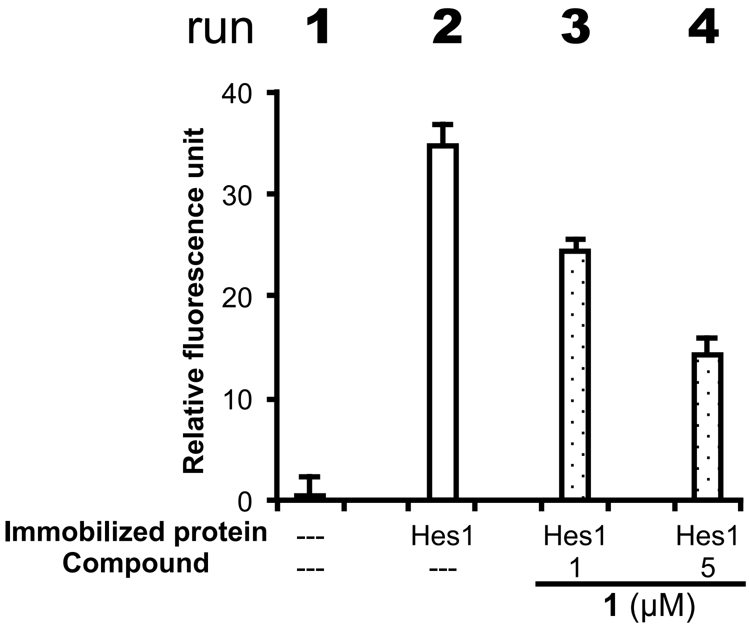

Figure S8-a. Hes1 dimer inhibition by lindbladione (**1**). Run 1; no immobilized protein, with Cy3-Hes1, run 2; immobilized Hes1 with Cy3-Hes1 after 24 h incubation, run 3, 4; Hes1 dimer inhibition by **1**.

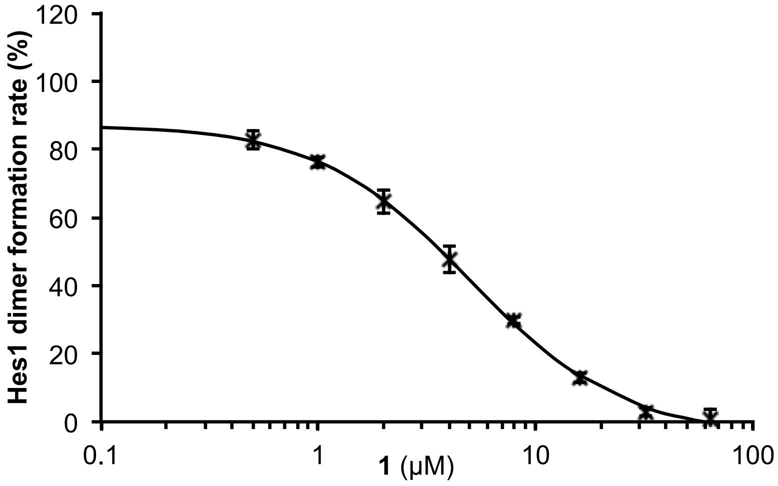

Compound	IC_{50} (μM)
**1**	3.7

Figure S8-b. Hes1 dimer inhibition by lindbladione (**1**). The background value (without Hes1, Cy3-Hes1 treated) was subtracted.

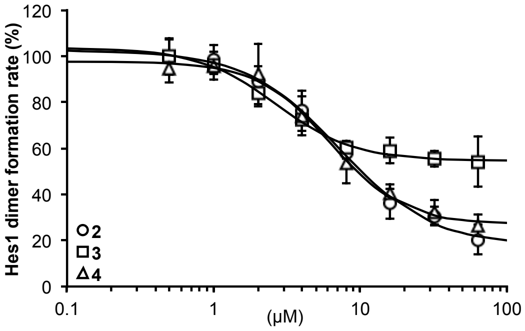

Compound	IC_{50} (μM)
**2**	10.1
**3**	> 64
**4**	9.5

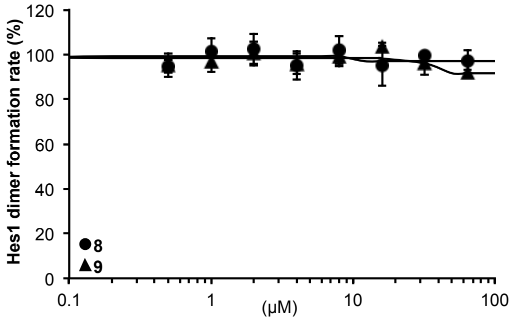

Compound	IC_{50} (μM)
**8**	> 64
**9**	> 64

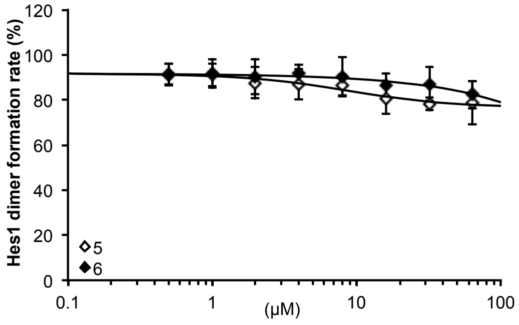

Compound	IC_{50} (μM)
**5**	> 64
**6**	> 64

Figure S8-c. Hes1 dimer inhibition by compounds. The background value (without Hes1, Cy3-Hes1 treated) was subtracted.

Cell culture

Mouse embryo fibroblast cells (C3H10T1/2) and mouse neural stem cells (MEB5) were purchased from Health Science Research Resources Bank (HSRRB). C3H10T1/2 cells were cultured in Dulbecco's modified Eagle Medium (DMEM, high glucose, Wako) that containing 10% fetal bovine serum (FBS, biowest). MEB5 cells were cultured and propagated as floating neurospheres in "proliferation medium" (DMEM (high glucose and HEPES, Wako) supplemented with 5 mg L^{-1} Insulin (Invitrogen), 10 μg L^{-1} Epidermal Growth Factor (rh EGF, VERITAS), 50 mg L^{-1} Transferin (Holo form, GIBCO), 10 μg L^{-1} (+)-Biotin (Wako) and 30 nM Na_2SeO_3 (Wako) or NeuroClut Basal Medium (Mouse, VERITAS) supplemented with 10% NSC Proliferation Supplements (Mouse, VERITAS)). For neural differentiation of MEB5, cells were transferred to "differentiation medium" (NeuroCult Basal Medium (Mouse) supplemented with 10% NSC Proliferation Supplements (Mouse, VERITAS)). All cultures were maintained in a humidified incubator at 37 ºC in 5% CO_2/95% air.

Co-immunoprecipitation assay

C3H10T1/2 cells (5×10^5 cells) were transfected with pCI-HA-Hes1 (1.5 μg/dish) and pCI-FLAG-Hes1 (1.5 μg/dish) using Lipofectamine 2000 (20 μL/dish, Invitrogen) and Opti-MEM (480 μL/dish, Gibco) in 60 mm dish. After 3 h transfection, the medium was changed to the fresh medium containing each compounds and the cells were incubated

for 24 h. After 24 h, the cell lysis was performed in the binding buffer (20 mM Tris-HCl, pH 7.5, 150 mM NaCl, 1 mM EDTA and 0.3% Nonidet P-40) supplemented with 1% of protease inhibitors cocktail (Nacalai tesque Inc.) Each cell lysate was incubated with anti-HA agarose besds (bed volume 20 μL, Sigma Aldrich) overnight with rotary mixing at 4ºC. After washing, bound proteins were eluted with 2 × SDS-PAGE sample buffer (125 mM Tris-HCl, pH 6.8, 4% SDS, 10% sucrose, 0.01% bromo phenol blue, 8% 2-mercaptoethanol) and the supernatant was subjected to a 12% SDS-PAGE, then transferred to a polyvinylidine difluoride (PVDF) membrane (BIO-RAD). After blocking with TBST (10 mM Tris-HCl, pH 7.4, 100 mM NaCl and 0.01% Tween 20) containing 5% ECL Prime Blocking Reagent (GE Healthcare) overnight at 4ºC, the blots were incubated at RT for 1 h with primary antibodies. After washing with TBST, the blots were incubated at RT for 1 h with secondary antibodies that conjugated with horseradish peroxidase (HRP). After washing, the immnocomlexed bands were detected using ECL Advance Western detection system (GE Healthcare) or an Immobilon Western detection system (Millipore). The analysis was performed by using antibodies to rabbit anti-HA (1/500 or 1/1000, Sigma Aldrich), rabbit anti-FLAG (1/1000 or 1/2000, Sigma Aldrich) and goat anti-HES1 (1/200, Santa Cruz Biotechnology) as primary antibodies, and goat anti-rabbit IgG (1/10000, Jackson Immuno Research) and donkey anti-goat IgG (1/10000, Jackson Immuno Research) as secondary antibodies. The quantity of each protein was measured by Molecular Imager ChemiDoc XRS+

(Bio-Rad Lab.), and is expressed as a ratio of control (without compound).

Differentiation assay

Floating neurospheres of MEB5 cells were dissociated using NeuroCult Chemical Dissociation Kit (STEMCELL) and plated on glass coverslips coated with the poly-L-lysine, fibronectin and laminine in the 24-well plates in proliferation medium at a density of 2×10^4 cells/well. After 12 h incubation, the cells were washed with NeuroCult Basal Medium (Mouse), then incubated in differentiation medium with compounds at each concentration for four days.

Immunofluorescence staining

MEB5 cells were fixed with 4% paraformaldehyde in PBS for 20 min at RT and washed twice with PBS containing 1% BSA. The fixed cells were permeablilized and blocked with 0.3% Triton X-100 and 10% BSA in PBS for 45 min at RT and incubated overnight at 4ºC with primary antibodies. After washing with PBS containing with 1% BSA, the cells were incubated at RT for 1 h with secondary antibodies that were conjugated with fluorescent dyes. After washing, cells were incubated with 200 μg/mL RNase (Invitrogen) in PBS containing 1% BSA and 0.1% Triton X-100 for 1 h at 37ºC. After washing with PBS, cells were treated with 3 μM TO-PRO®-3 (Invitrogen) for 10 min (for nuclei staining) at RT under the dark. Finally, the cells were washed with PBS

and mounted with ProLong® Gold Antifade Reagent with DAPI (Invitrogen). Fluorescence was detected and photographed at RT with a LSM700 confocal laser scaning microscope (Carl Zeiss). Total nine pictures were taken per each well and the assays were carried out in three individual wells. The analysis was performed by using antibodies to mouse anti-βIII-Tubulin (neuron marker, 1/400, R&D) and rabbit anti-GFAP (astrocyte marker, 1/400, VERITAS) as primary antibodies, and Alexa Fluor 488 goat anti-mouse IgG (1/400, Invitrogen) and Alexa Fluor 555 goat ant-rabbit IgG (1/200, Invitrogen) as secondary antibodies. More than 3000 cells were counted and more than 800 neurite length were measured per each sample. Cell count and measurement of the neurite length were conducted using ImageJ software.

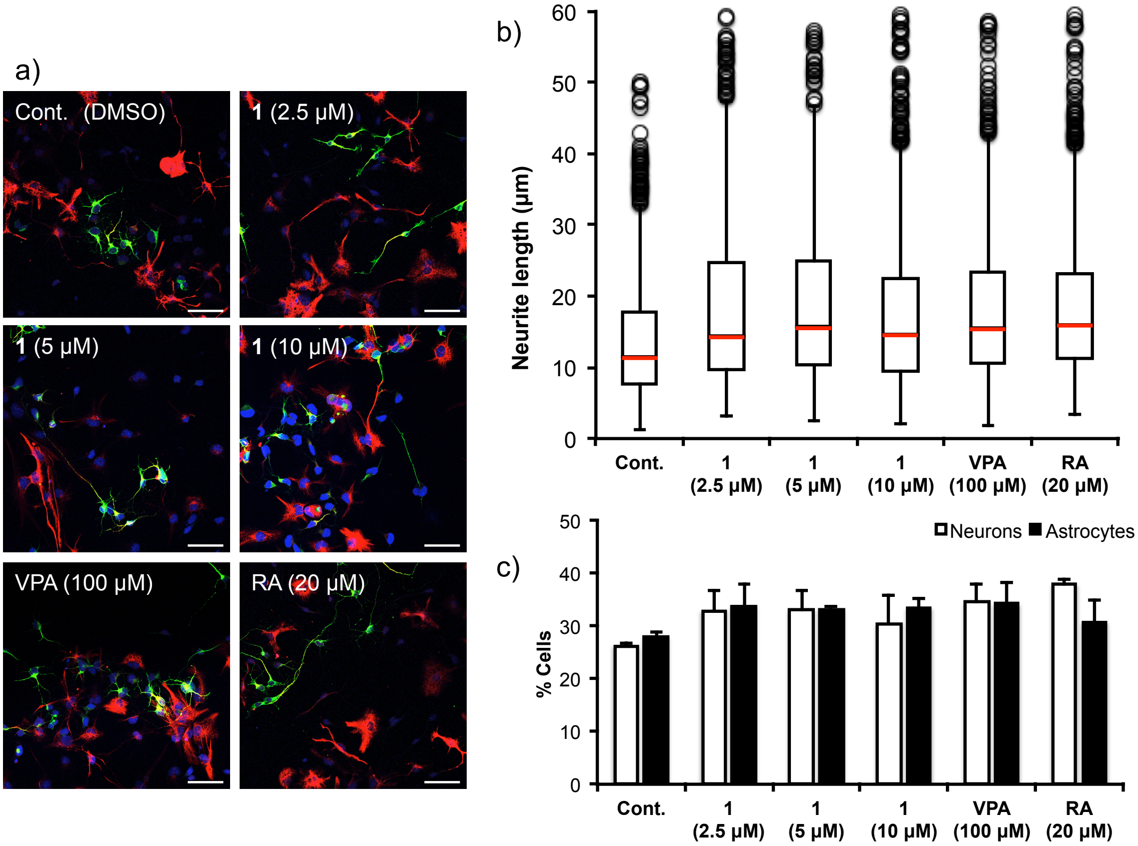

Figure S9. NSC differentiation-promoting activity of **1**. a) MEB5 cells were treated with DMSO (negative control), lindbladione (**1**) (2.5, 5.0, 10 μM), VPA (positive control; 100 μM) or RA (positive control; 20 μM) for four days. The cells were then immunostained with Tuj1 (green) for neurons, GFAP (red) for astrocytes, and TO-PRO-3 (blue) for nuclei. Scale bar: 50 μm. DMSO = dimethylsulfoxide, VPA = valproic acid, RA = retinoic acid. VPA and RA were positive controls. b) Effects on the length of neurites. The lengths of the neurites are shown as box plots: middle bars (red) show the meadian values, each box and bar shows 25% of total number of neurites. c) Effects on the number of neurons and astrocytes.

RNA isolation and RT-PCR analysis

MEB5 cells (4×10^5 cells) in the proliferation medium were seeded onto the poly-L-lysine/fibronectin/laminin-coated 10 cm dish and cultured at 37ºC for 12 h. After the medium was changed to the differentiation medium including compound, the cells were incubated for 24-96 h. Total RNA was isolated from the cells using an RNeasy Mini Kit (QIAGEN) according to the manufacturer's protocol. cDNA was synthesized using Superscript® VILO™ (Invitrogen). Real-time PCR reactions were performed using SYBR® GreenER™ qPCR SuperMix Universal (Invitrogen). The reactions were performed using Mx3000P QPCR System (Stratagene), using following program: 50 ºC for 2 min (initial incubation), 95 ºC for 10 min (initial denaturation) and then 40 cycles of 95 ºC for 15 s (denaturation) and 60 ºC for 60 s (annealing and extension). Sequences of the primers were listed in **Table S2**. A fluorescence signal was collected at the end of each cycle. After the reactions were terminated, the signal at each temperature from 55 to 95 ºC was also collected for dissociation curve analysis. All reactions were performed in triplicate to confirm reproducibility, and the amount of target mRNA in each sample was normalized with that of the mean GAPDH.

Table S2. Oligonucleotides primers used for RT-PCR analysis in this work.

Genes	Primers	Sequences
mouse *Mash1*	Mash1-F	5'-TCTCCTGGGAATGGACTTTG-3'
	Mash1-R	5'-GGTTGGCTGTCTGGTTTGTT-3'
mouse *Ngn2*	Ngn2-F	5'-GCTGTGGGAATTTCACCTGT-3'
	Ngn2-R	5'-CCTGGCCCTCTAACAAAACA-3'
mouse *NeuroD2*	NeuroD2-F	5'-TGCCCTGCTACTCCAAGAC-3'
	NeuroD2-R	5'-CCGTGAGGAAGTTACGAGAGTT-3'
mouse *GAPDH*	GAPDH-F	5'-GCACAGTCAAGGCCGAGAAT-3'
	GAPDH-R	5'-GCCTCCTCCATGGTGGTGAA-3'

ELISA for inhibitors of TCF/β-catenin complex formation

Nunc Immobilizer^{TM} Amino 96 well plate was used for immobilizing of hTCF4E$_{1\text{-}100}$. The microplate wells were incubated with 100 μL of hTCF4E$_{1\text{-}100}$ (5 mg L^{-1} in PBS) for 2 h at 4ºC. After the removal of protein solution, to block the well, the wells were incubated for 2 h at 4ºC with 100 μL of 10 mM ethanolamine, then washed twice with 200 μL of PBST. The TCF bound microplate wells were incubated with 50 μL of GST-mβ-catenin (20 mg L^{-1}) for 30 min at 4ºC. After removal of protein solution, each well was washed twice with 200 μL of PBST, then each compound solution (NET-N buffer, 50 μL) was added. After incubation for 1 h at RT in the dark, each well was washed twice with 200 μL of PBST. To detect GST-β-catenin which bound on TCF, the wells were incubated with 50 μL of HRP conjugated anti-GST antibody (1 μg L^{-1}, Bethyl Laboratories, Inc.) for 1 hr at RT under the dark. After washing twice with PBST, the wells were treated with 50 μL of SuperSignal ELISA Femto Maximum Sensitivity

Substrate (Thermo), then the luminescence activity was measured in a microplate luminometer (Luminoskan Ascent, Thermo). The assays were carried out in three individual wells, and the mean value and SD were calculated.

This assay was constructed by PKF115-584 as a positive control (Figure S10). (Interestingly, in the dark, PKF115-584 didn't inhibit the complex.)

The results of TCF/β-catenin complex formation inhibition were shown (Figure S11).

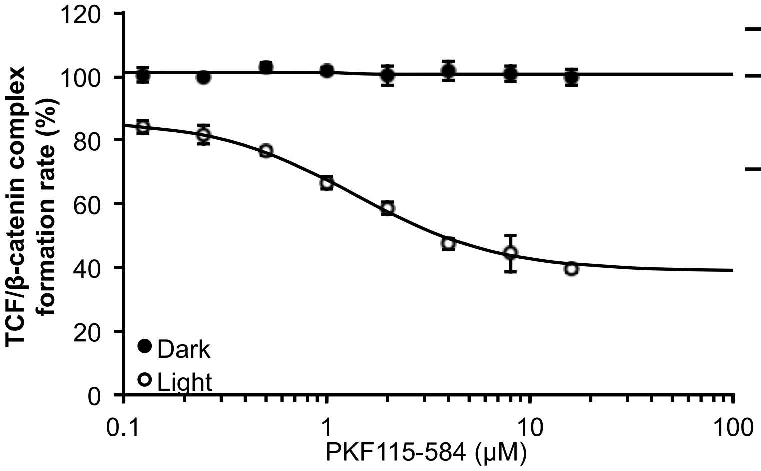

Conditions	IC_{50} (μM)
**In dark**	> 64
**Under the light**	3.6

Figure S10. ELISA for inhibitors of TCF/β-catenin complex; black; in dark, white; under the light.

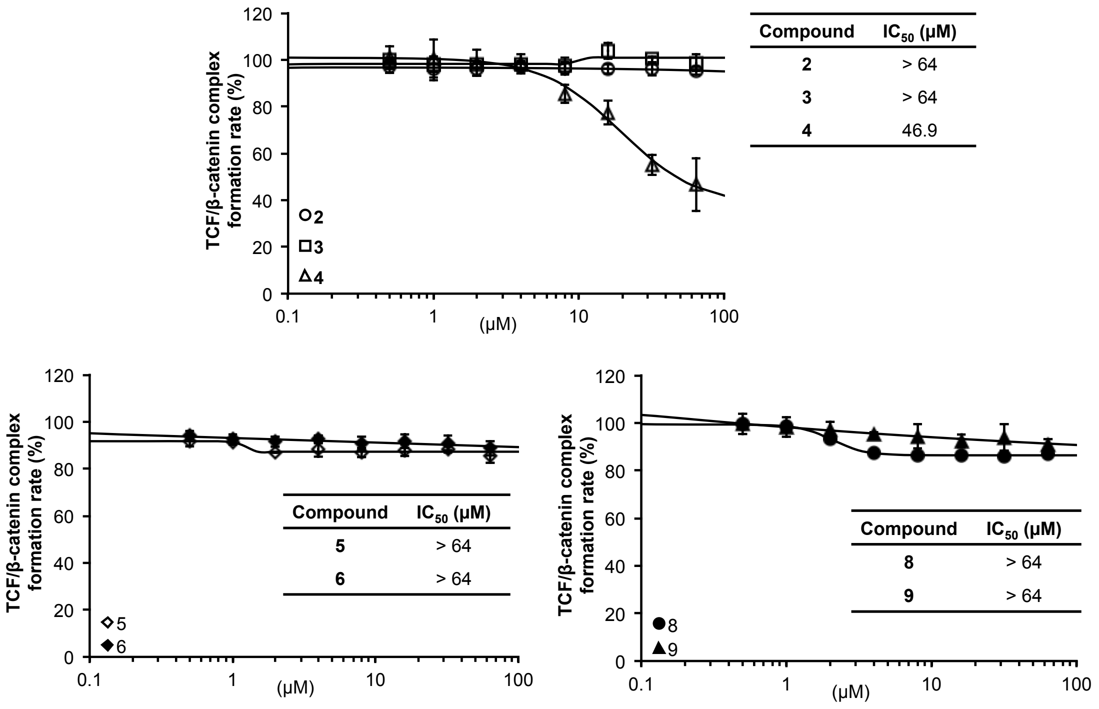

Figure S11. ELISA for inhibitors of TCF/β-catenin complex.

Inhibition of N-box dependent repression activity by Hes1 dimers

C3H10T1/2 cells (5×10^4 cells/well) were transfected with plasmids using Lipofectamine 2000 (2 µL/well) and Opti-MEM (48 µL) in 24-well plate for 3 h. The following plasmids were used for transfection: a reporter plasmid pN6-βA-luc (0.1 µg/well) containing the six N-box sequence (CACNAG) was cotransfected with 0.3 µg of pCI-Hes1. As a reference plasmid for normalization, 2 ng of pRL-SV40 plasmid (Promega) was used. After 3 h transfection, the medium was changed to the fresh medium containing each compounds and the cells were incubated for 24 h. After 24 h, the cells were harvested and luciferase activity was measured in a microplate luminometer using Pick-A-Gene® Dual (TOYO B-Net) according to the manufacturer's

protocol. The mean value and SD of three individual wells were calculated.

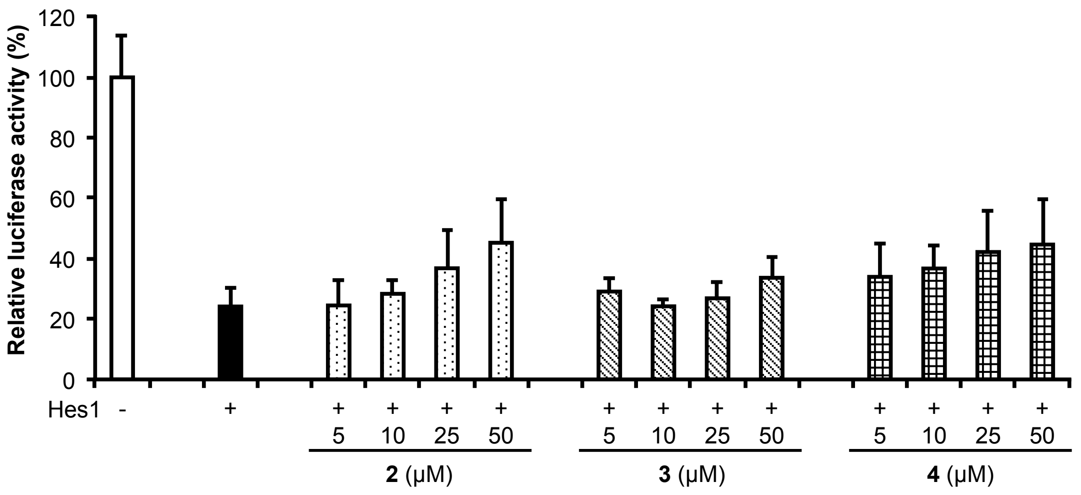

Figure S12. Hes1-dependent reporter gene assay. Compounds **2** and **4** cancelled the N-box dependent repression activity by Hes1 dimers dose dependent manner.

Molecular modeling calculations

All molecular modeling calculations were conducted by Spartan 10 software (Wavefunction). The stable conformers of compound **2**, **3** and **4** were obtained using molecular mechanism calculation to afford 15, 16 and 19 stable conformers, respectively. The conformers with a relative energy of 0-5 kJ mol^{-1}, eleven, ten and two conformers for compound **2**, **3** and **4**, respectively, were optimized using Hartree-Fock/3-21G calculation followed by DFT methods supporting no solvent (vacuum conditions, B3LYP/6-31G*). Lowest energy (global minimum) structures were presented in **Figure S13**. Relative energy for local minimum conformers were 0, 0.22, 1.84, 2.30, 2.33, 2.35, 2.74, 3.68, 3.72, 4.83 and 4.86 kJ mol^{-1} for compound **2**, 0,

0.02, 1.28, 1.43, 1.49, 2.72, 2.75, 3.65, 3.74 and 4.91 kJ mol^{-1} for compound **3** and 0 and 4.01 kJ mol^{-1} for compound **4**, respectively. Interestingly, only agalloside (**2**) has obvious electron deficient area (**Figure S12**).

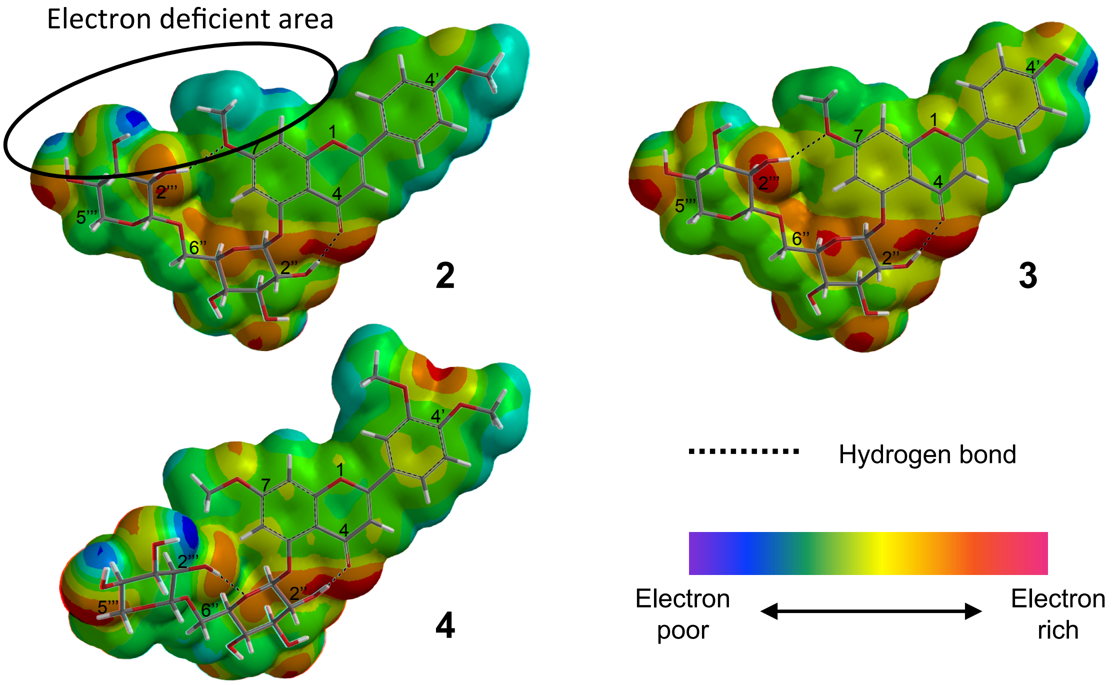

Supporting Figure S13. Electrons Density Distribution by DFT calculation. Agalloside (**2**) has obvious electron deficient area.

Atomic coordinates for compound **2**

Atom	X	Y	Z
C	-0.986021	2.391980	-0.096159
C	-2.216288	4.445187	0.519924
C	-3.310896	2.222462	0.227284
C	-3.506293	3.739507	0.085065
C	-0.993374	3.911177	-0.224671
O	0.079804	1.918493	-0.877351
O	0.158903	4.491134	0.349251
O	-2.406432	5.839154	0.310863

O	-4.597572	4.146266	0.893986
O	-2.193675	1.843683	-0.581124
C	-4.489115	1.378843	-0.214344
O	-4.173128	0.020362	0.111143
C	-5.137533	-0.903709	-0.266425
C	-5.708485	-3.333125	-0.283978
C	-7.374897	-1.563334	0.210803
C	-6.972312	-3.010035	0.502525
C	-4.599325	-2.319714	-0.004577
O	-6.302296	-0.681276	0.523222
O	-3.518072	-2.686816	-0.852665
O	-5.301784	-4.654109	0.051144
O	-8.046202	-3.859156	0.130672
H	0.946990	4.028467	-0.010968
H	-1.084358	4.151645	-1.296006
H	-0.835711	2.096321	0.955717
H	-1.562768	6.265845	0.535373
H	-2.070410	4.244251	1.594807
H	-4.544158	5.115759	0.942952
H	-3.693963	3.973283	-0.975546
H	-3.104059	1.989628	1.284299
H	-4.641176	1.479096	-1.299305
H	-5.393415	1.702507	0.308458
H	-5.393073	-0.789884	-1.338000
H	-4.313103	-2.372983	1.055456
H	-2.682367	-2.348034	-0.490150
H	-5.954273	-3.278873	-1.358603
H	-4.460101	-4.787514	-0.416284
H	-7.693704	-4.764070	0.158384
H	-6.748694	-3.107483	1.577082
H	-8.220536	-1.260660	0.832448
H	-7.672473	-1.471013	-0.846263
C	0.576203	0.684341	-0.572255
C	1.649194	-1.857987	-0.089612
C	1.978280	0.571472	-0.393841
C	-0.264430	-0.410403	-0.479220
C	0.274446	-1.678471	-0.218263
C	2.470514	-0.727878	-0.184636
O	3.808824	-0.977397	-0.046809
C	2.909830	1.720412	-0.332505

C	4.307502	1.335150	-0.202546
C	4.713856	0.045502	-0.078606
C	6.096465	-0.431861	0.058898
C	8.750461	-1.341738	0.350071
C	6.369187	-1.734583	0.521603
C	7.184074	0.392870	-0.264686
C	8.497428	-0.046178	-0.122080
C	7.671650	-2.182677	0.666940
O	9.987653	-1.873472	0.527529
O	-0.649862	-2.675256	-0.127121
C	11.123066	-1.064397	0.247591
C	-0.210191	-4.016915	0.061134
H	11.138161	-0.165354	0.876342
H	11.992007	-1.682634	0.477969
H	11.155855	-0.769699	-0.809030
H	9.309218	0.620229	-0.388813
H	7.883623	-3.183734	1.028689
H	5.546954	-2.394110	0.774627
H	7.011530	1.391886	-0.652444
H	5.027011	2.142961	-0.163727
H	-1.331582	-0.288018	-0.616568
H	2.105901	-2.823637	0.084088
H	0.323751	-4.128211	1.012443
H	0.433809	-4.340539	-0.765117
H	-1.118671	-4.619894	0.075727
O	2.577868	2.913046	-0.354458

Atomic coordinates for compound **3**

Atom	X	Y	Z
C	-0.644470	-2.379099	0.087835
C	-1.832798	-4.462706	-0.510779
C	-2.972756	-2.260513	-0.231597
C	-3.135243	-3.780368	-0.075666
C	-0.619288	-3.897555	0.225895
O	0.412626	-1.880368	0.865512
O	0.543993	-4.455893	-0.347582
O	-1.993382	-5.858742	-0.290627
O	-4.222345	-4.218001	-0.873508

O	-1.861999	-1.851771	0.571242
C	-4.168865	-1.440252	0.206492
O	-3.882668	-0.075788	-0.121876
C	-4.858216	0.830361	0.271039
C	-5.477493	3.249130	0.288518
C	-7.116296	1.444902	-0.167399
C	-6.747968	2.897426	-0.474428
C	-4.353124	2.256194	-0.002854
O	-6.032441	0.582700	-0.496710
O	-3.267979	2.647814	0.829626
O	-5.101423	4.575781	-0.060085
O	-7.831014	3.728040	-0.088094
H	1.322949	-3.976771	0.011090
H	-0.702223	-4.132704	1.299187
H	-0.500978	-2.085995	-0.965809
H	-1.143517	-6.270520	-0.519546
H	-1.694675	-4.266885	-1.587560
H	-4.150589	-5.186847	-0.910185
H	-3.311978	-4.007212	0.988305
H	-2.772604	-2.030939	-1.290679
H	-4.320098	-1.541187	1.291448
H	-5.065344	-1.785473	-0.316131
H	-5.092168	0.714133	1.347202
H	-4.082221	2.311937	-1.066669
H	-2.430509	2.323496	0.458300
H	-5.704778	3.196249	1.367229
H	-4.257013	4.728736	0.396152
H	-7.495589	4.639114	-0.124781
H	-6.545604	2.992681	-1.553305
H	-7.967641	1.122275	-0.771116
H	-7.390492	1.352656	0.896074
C	0.885114	-0.638512	0.555469
C	1.912294	1.921851	0.068232
C	2.285773	-0.499678	0.382832
C	0.024152	0.439499	0.453261
C	0.540378	1.716570	0.189562
C	2.754154	0.807901	0.171117
O	4.088420	1.082207	0.039054
C	3.239238	-1.630769	0.329287
C	4.630186	-1.219851	0.202643

C	5.012106	0.076725	0.076619
C	6.386172	0.580049	-0.056892
C	9.016005	1.544384	-0.340401
C	6.634449	1.884895	-0.523145
C	7.488977	-0.224706	0.275343
C	8.788406	0.246449	0.134760
C	7.928871	2.364019	-0.666356
O	10.264263	2.063754	-0.499444
O	-0.401809	2.695138	0.089179
C	0.013821	4.044191	-0.100928
H	9.628345	-0.390997	0.403777
H	8.118860	3.368277	-1.031102
H	5.799227	2.525518	-0.781700
H	7.336215	-1.225787	0.665670
H	5.364523	-2.014528	0.169622
H	-1.041044	0.297248	0.587778
H	2.351673	2.895468	-0.105079
H	0.647573	4.381797	0.727578
H	-0.905229	4.630757	-0.121725
H	0.550501	4.162690	-1.049842
O	2.930705	-2.829430	0.354662
H	10.924305	1.398200	-0.249444

Atomic coordinates for compound **4**

**Atom**	**X**	**Y**	**Z**
C	1.709504	-2.036392	0.291744
C	2.816366	-4.229497	0.653310
C	4.073328	-2.069215	0.621133
C	4.145287	-3.572111	0.304853
C	1.672056	-3.524922	-0.060373
O	0.701534	-1.420400	-0.468380
O	0.473772	-4.133379	0.354528
O	2.898409	-5.600902	0.294736
O	5.212022	-4.130154	1.056390
O	2.965192	-1.463831	-0.068550
C	5.337003	-1.349208	0.180646

O	5.195270	0.028103	0.511509
C	5.728655	0.966106	-0.364980
C	5.341366	3.271724	-1.227536
C	7.607023	2.343762	-0.761056
C	6.755617	3.618444	-0.770553
C	4.773521	2.162044	-0.355015
O	7.022605	1.341615	0.070970
O	3.495384	1.793238	-0.861901
O	4.565424	4.465216	-1.172050
O	7.365018	4.553223	-1.644692
H	-0.267462	-3.709345	-0.141004
H	1.817167	-3.602075	-1.149604
H	1.519644	-1.885008	1.365615
H	1.992209	-5.945879	0.372137
H	2.658002	-4.133164	1.741412
H	5.105154	-5.094358	0.994819
H	4.321794	-3.698235	-0.776126
H	3.943728	-1.941452	1.707051
H	5.466213	-1.467585	-0.903826
H	6.201032	-1.788020	0.689757
H	5.784720	0.552619	-1.389771
H	4.696883	2.517364	0.683704
H	3.321413	0.879110	-0.567366
H	5.396305	2.905176	-2.265820
H	3.683486	4.243384	-1.511586
H	6.712358	5.262472	-1.769922
H	6.701114	4.017764	0.254916
H	7.722795	1.979497	-1.794722
H	8.600376	2.544554	-0.354630
C	-0.080130	-0.448097	0.091417
C	-1.701360	1.629799	1.019248
C	-1.483311	-0.579493	-0.049947
C	0.500149	0.653865	0.709027
C	-0.315925	1.698544	1.176864
C	-2.253582	0.510222	0.411143
O	-3.617327	0.531230	0.292126
C	-2.181584	-1.767186	-0.582722
C	-3.618961	-1.609449	-0.711211
C	-4.288072	-0.507833	-0.281479
C	-5.738119	-0.275739	-0.333104

C	-8.522915	0.181909	-0.394928
C	-6.598313	-1.186949	-0.952205
C	-6.290940	0.882224	0.256735
C	-7.658665	1.119110	0.231458
C	-7.974488	-0.959950	-0.980342
O	-9.842525	0.490155	-0.367761
O	0.154941	2.821084	1.766024
C	-10.756506	-0.414852	-0.970913
C	1.566981	2.979024	1.910792
H	-10.726151	-1.398924	-0.485684
H	-11.744719	0.026742	-0.833589
H	-10.556204	-0.532450	-2.043730
H	-8.617143	-1.685202	-1.465776
H	-6.211010	-2.082888	-1.424452
H	-5.629555	1.589188	0.737631
H	-4.153550	-2.451918	-1.129549
H	1.577656	0.711972	0.767641
H	-2.332079	2.443127	1.357085
H	2.072674	2.940468	0.939935
H	1.984715	2.210416	2.573226
H	1.704465	3.961247	2.364801
O	-1.642165	-2.842764	-0.890451
O	-8.271681	2.204279	0.776253
C	-7.462196	3.170155	1.429028
H	-6.732352	3.616534	0.741051
H	-8.148287	3.942496	1.780634
H	-6.931169	2.736440	2.286610

The binding region of agalloside (2) in Hes1.

To predict the binding region of compound **2** in Hes1, Hes1 beads assay method using partial proteins of Hes1 was performed described above (see a typical screening procedure). Briefly, compound **2** (12.5 nmol in DMSO) was mixed and incubated with full and partial Hes1 beads (ca. 3.6 nmol each) and control GST beads (ca. 3.8 nmol).

The quantity of **2** bound to each protein was measured and calculated from the peak area of **2**, and the predicted binding ability is expressed as a ratio of GST-Hes1 (1-281 aa, Full) beads.

The docking model of agalloside (2) and HLH domain.

The initial 3D structures of the ligands were constructed using the BUILDER module in the molecular modeling program Insight II (www.accelrys.com) with standard geometric parameters. The ligand was then minimized in the DISCOVER module in Insight II with Amber force field until the root-mean-squares (rms) energy gradient was less than 0.005 kcal $mol^{-1}Å^{-1}$.

The protein model was constructed based on the structure of HLH domain taken from the Protein Data Bank (PDB) (PDB code: 2MH3). (*Proteins*, **2014**, *82*, 537-545). Water molecules in the crystal structure were removed. All hydrogen atoms were added and Amber all-atom charges were assigned for the whole protein.

The program GOLD (Genetic O ptimization for Ligand Docking) 3.0 was used for molecular docking simulations. The GOLD program uses a genetic algo rithm for docking flexible ligands into protein active site. The genetic parameters for docking were as follows: output of low energy conformation 10, population size 100, selection pressure 1.1, number of operation 100,000, number of islands 5, nich size 2, migra te 10, mutate 95, and crossover 95.

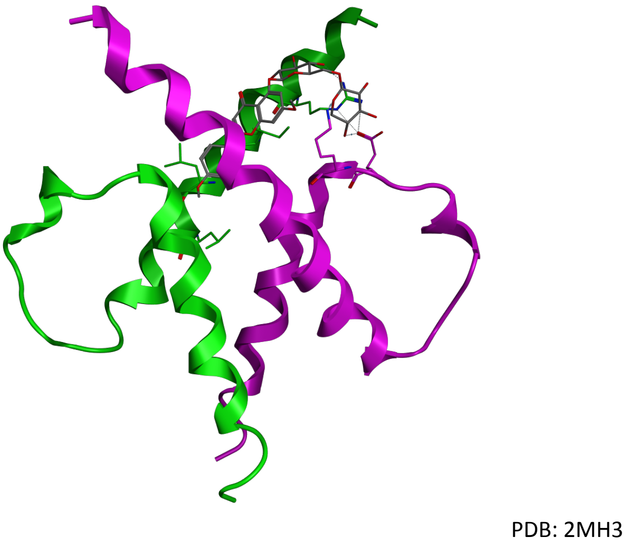

Supporting Figure S14a.

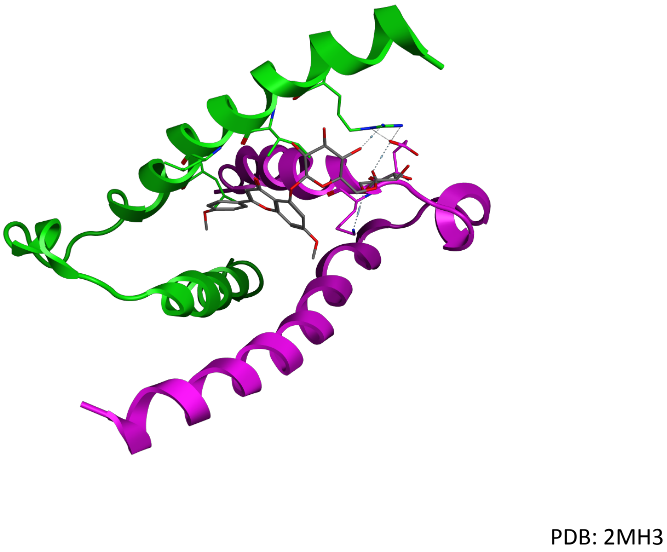

Supporting Figure S14b.

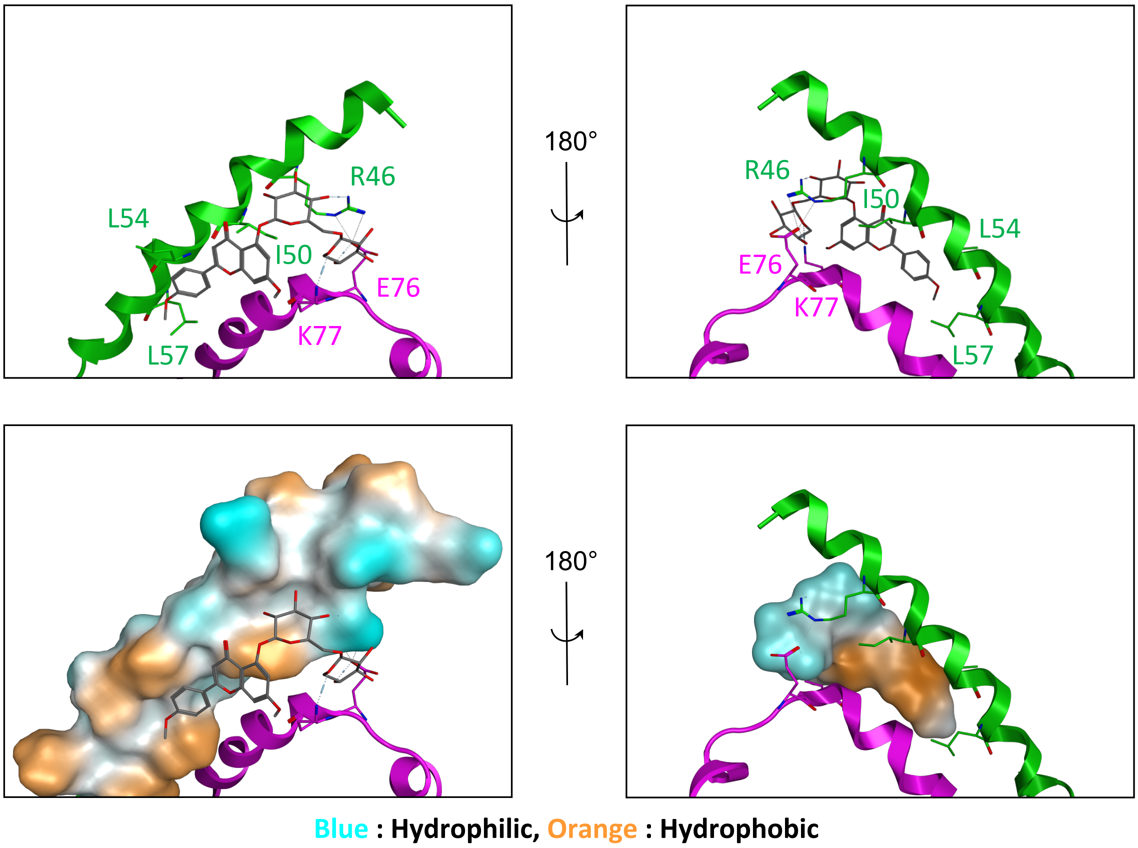

Supporting Figure S14c.

Spectroscopic data of isolated compounds.

7,4'-Di-*O*-methylapigenin 5-*O*-β-D-xylosyl-β-D-glucoside, Agalloside (2)

A yellow solid; $[\alpha]_D^{21}$ -66.6 (*c* 0.31, pyridine), $[\alpha]_D^{23}$ -43.2 (*c* 0.30 MeOH); ^{1}H NMR (DMSO-d_6, 400 MHz) 8.04 (2H, d, *J* = 9.0 Hz, H-2' and H-6'), 7.09 (2H, d, *J* = 9.0 Hz, H-3' and H-5'), 7.06 (1H, d, *J* = 2.0 Hz, H-8), 6.87 (1H, d, *J* = 2.0 Hz, H-6), 6.80 (1H, s, H-3), 4.78 (1H, d, *J* = 7.6 Hz, H-1''), 4.18 (1H, d, *J* = 7.6 Hz, H-1'''), 3.97 (1H, d, *J* = 10.7 Hz, H-6''), 3.89 (3H, s, OMe-7), 3.84 (3H, s, OMe-4'), 3.68 (1H, m, H-5'''), 3.65 (1H, dd, *J* = 10.7, 4.6 Hz, H-6''), 3.56 (1H, t, *J* = 8.0 Hz, H-3'''), 3.36 (1H, m, H-2'''), 3.31 (1H, m, H-5''), 3.28 (1H, t, *J* = 8.4 Hz, H-4''), 3.24 (1H, m, H-4'''), 3.09 (1H, t, *J* = 8.6 Hz, H-3''), 3.01 (1H, m, H-5''') and 2.98 (1H, m, H-2''); ^{13}C NMR (DMSO-d_6, 100 MHz) 176.9 (C-4), 163.7 (C-7), 162.1 (C-4'), 160.9 (C-2), 158.5 (C-9), 158.2 (C-5),

128.1 (C-2’ and C-6’), 122.8 (C-1’), 114.6 (C-3’ and C-5’), 109.2 (C-10), 106.5 (C-3), 104.1 (C-1’’’), 103.7 (C-1’’), 102.9 (C-6), 96.7 (C-8), 76.6 (C-3’’), 75.9 (C-3’’’), 75.6 (C-5’’), 73.5 (C-2’’’), 73.4 (C-2’’), 69.7 (C-4’’’), 69.5 (C-4’’), 68.9 (C-6’’), 65.7 (C-5’’’), 56.2 (OMe-7) and 55.6 (OMe-4’); ESI-MS: *m/z* 615 $[M+Na]^+$.

Genkwanin 5-*O*-β-D-xylosyl-β-D-glucoside (3)

A white needle crystal; $[\alpha]_D^{22}$ -52.3 (*c* 0.31, pyridine), $[\alpha]_D^{24}$ -28.1 (*c* 0.30 MeOH); ^{1}H NMR (DMSO-d_6, 400 MHz) 7.92 (2H, d, *J* = 8.8 Hz, H-2’ and H-6’), 7.03 (1H, d, *J* = 2.2 Hz, H-8), 6.91 (2H, d, *J* = 8.8 Hz, H-3’ and H-5’), 6.86 (1H, d, *J* = 2.2 Hz, H-6), 6.71 (1H, s, H-3), 4.77 (1H, d, *J* = 7.2 Hz, H-1’’), 4.18 (1H, d, *J* = 7.6 Hz, H-1’’’), 3.97 (1H, d, *J* = 10.8 Hz, H-6’’), 3.89 (3H, s, OMe-7), 3.70 (1H, m, H-5’’’), 3.65 (1H, dd, *J* = 10.2 Hz, H-6’’), 3.56 (1H, t, *J* = 8.8 Hz, H-5’’), 3.39 (1H, t, *J* = 8.8 Hz, H-2’’), 3.36 (1H, t, *J* = 8.8 Hz, H-3’’), 3.29 (1H, t, *J* = 8.8 Hz, H-4’’), 3.22 (1H, t, *J* = 8.8 Hz, H-4’’’), 3.09 (1H, t, *J* = 8.8 Hz, H-3’’’), 3.02 (1H, d, *J* = 10.2 Hz, H-5’’’) and 2.96 (1H, t, *J* = 8.4 Hz, H-2’’’); ESI-MS: *m/z* 601 $[M+Na]^+$.

Lethedioside A (4)

A yellow solid; $[\alpha]_D^{21}$ -29.4 (*c* 0.31, pyridine), $[\alpha]_D^{20}$ -24.2 (*c* 0.30 MeOH); ^{1}H NMR (pyridine-d_5, 400 MHz) 7.63 (1H, dd, *J* = 8.4, 1.6 Hz, H-6’), 7.50 (1H, d, *J* = 1.8 Hz, H-6), 7.06 (1H, d, *J* = 8.4 Hz, H-5’), 6.95 (1H, s, H-3), 6.94 (1H, d, *J* = 1.8 Hz, H-8), 5.36 (1H, d, *J* = 7.2 Hz, H-1’’), 4.96 (1H, d, *J* = 7.2 Hz, H-1’’’), 4.86 (1H, d, *J* = 10.8

Hz, H-6’’), 4.39 (1H, t, J = 8.0 Hz, H-2’’), 4.36, (1H, m, H-5’’), 4.34 (1H, m, H-5’’’), 4.32 (1H, t, J = 8.8 Hz, H-3’’), 4.31 (1H, m, H-6’’), 4.22 (1H, t, J = 9.0 Hz, H-4’’), 4.21 (1H, m, H-4’’’), 4.14 (1H, t, J = 8.6 Hz, H-3’’’), 4.04 (1H, t, J = 8.0 Hz, H-2’’’), 3.87 (3H, s, OMe-3’), 3.81 (3H, s, OMe-7), 3.78 (3H, s, OMe-4’) and 3.68 (1H, d, J = 10.7 Hz, H-5’’’); ^{13}C NMR (pyridine-d_5, 100 MHz) 178.0 (C-4), 164.5 (C-7), 161.9 (C-2), 159.7 (C-5), 159.2 (C-9), 152.8 (C-4’), 149.3 (C-3’), 124.1 (C-1’), 120.3 (C-6’), 112.1 (C-5’), 110.6 (C-10), 109.9 (C-2’), 107.9 (C-3), 106.3 (C-1’’’), 106.1 (C-1’’), 104.7 (C-6), 97.4 (C-8), 78.3 (C-3’’’), 77.9 (C-3’’), 77.7 (C-5’’), 75.2 (C-2’’), 75.0 (C-2’’’), 71.4 (C-4’’), 71.1 (C-4’’’), 70.3 (C-6’’), 67.2 (C-5’’’), 56.1 (OMe-3’), 56.0 (OMe-7) and 55.9 (OMe-4’); ESI-MS: *m/z* 645 $[M+Na]^+$.

α-Mangostin (5)

A yellow solid; ^{1}H NMR ($CDCl_3$, 400 MHz): 6.81 (1H, s, H-5), 6.28 (1H, s, H-4), 5.27 (1H, tsep, J = 7.4, 1.2 Hz, H-2’), 5.24 (1H, tsep, J = 6.2, 1.2 Hz, H-2’’), 4.07 (2H, d, J = 6.2 Hz, H-1’’), 3.79 (3H, s, OMe-7), 3.43 (2H, d, J = 7.4 Hz, H-1’), 1.82 (3H, d, J =1.2 Hz, H-4’), 1.81 (3H, d, J = 1.2 Hz, H-4’’), 1.75 (3H, d, J = 1.2 Hz, H-5’) and 1.67 (3H, d, J = 1.2 Hz, H-5’’); ESI-MS: *m/z* 409 $[M-H]^-$.

BE-14106 (6)

A white solid; ^{1}H NMR (DMSO-d_6, 600 MHz) 7.25 (1H, d, J = 10.2 Hz, NH), 6.64 (1H,

ddd, J = 15.0, 7.8, 2.5 Hz, H-3), 6.15 (1H, dd, J = 15.0, 11.4 Hz, H-16), 6.13 (1H, m, H-5), 6.12 (1H, m, H-4), 6.06 (1H, d, J = 15.2 Hz, H-13), 6.00 (1H, dd, J = 10.9, 10.9 Hz, H-11), 5.90 (1H, dd, J = 15.2, 10.9 Hz, H-12), 5.89 (1H, d, J = 15.0 Hz, H-2), 5.81, (1H, d, J = 11.4 Hz, H-15), 5.45 (1H, dd, J = 15.0, 7.0 Hz, H-22), 5.41 (1H, m, H-17), 5.40 (1H, m, H-21), 5.37 (1H, m, H-10), 5.16 (1H, d, J = 7.7 Hz, H-7), 4.98 (1H, brs, OH-9), 4.85 (1H, brs, OH-8), 4.54 (1H, dd, J = 7.7, 2.0 Hz, H-8), 4.29 (1H, dd, J = 8.9, 2.0 Hz, H-9), 3.80 (1H, m, H-19), 2.31 (1H, ddd, J = 11.3, 5.4, 2.7 Hz, H-18), 2.12 (2H, dd, J = 6.3, 6.3 Hz, H-20), 1.93 (2H, dt, J = 7.0, 7.0 Hz, H-23), 1.79 (3H, s, H-26), 1.75 (1H, ddd, J = 11.3, 11.3, 11.3 Hz, H-18), 1.57 (1H, s, H-27), 1.32 (2H, tq, J = 7.0, 7.0 Hz, H-24) and 0.83 (3H, t, J = 7.0 Hz, H-25); ^{13}C NMR (DMSO-d_6, 150 MHz) 166.5 (C-1), 142.6 (C-5), 139.6 (C-3), 138.8 (C-7), 136.4 (C-13), 133.0 (C-14), 131.8 (C-22), 131.6 (C-6), 131.3 (C-10), 130.8 (C-16), 130.4 (C-15), 130.0 (C-17), 128.6 (C-11), 127.2 (C-21), 125.0 (C-4), 124.0 (C-2), 123.8 (C-12), 72.2 (C-8), 69.7 (C-8), 49.4 (C-19), 38.0 (C-20), 39.5 (C-18), 34.1 (C-23), 22.1 (C-24), 13.5 (C-25), 12.4 (C-26) and 12.0 (C-27); ESI-MS: *m/z* 424 $[M+H]^+$, *m/z* 847 $[2M+H]^+$, *m/z* 869 $[2M+Na]^+$ and *m/z* 422 $[M-H]^-$.

Inohanamine (7)

A yellow solid; $[\alpha]_D^{21}$ +28.1 (*c* 1.0, $CHCl_3$); IR (ATR) ν_{max} 3367, 2927, 1559, 1408, 1123 and 995 cm^{-1}; UV (MeOH) λ_{max} 257, 267 and 277 nm; ^{1}H and ^{13}C NMR, see Table

S1; HR-ESI-MS: m/z 208.1732 $[M+H]^+$ (calcd for $C_{13}H_{22}NO$ 208.1701).

Table S3. ^1H and ^{13}C NMR of inohanamine (**7**)

position	^1H NMR in $CDCl_3$ [ppm] (600 MHz)	^{13}C NMR in $CDCl_3$ [ppm] (150 MHz)
2	3.19 (dd, 9.8, 9.0 Hz, 1H)	59.1
3	1.53 (dqd, 9.0, 7.2, 3.0 Hz, 1H)	40.1
4	3.91 (ddd, 3.0, 3.0, 2.4 Hz, 1H)	69.0
5	1.80 (m, 2H)	33.2
6	2.86 (dt, 11.8, 3.6, 3.6 Hz, 1H)	39.9
6	3.08 (ddd, 11.8, 11.5, 4.0 Hz, 1H)	39.9
7	5.53 (dd, 15.7, 9.0 Hz, 1H)	133.9
8	6.18 (dd, 15.7, 10.5 Hz, 1H)	132.7
9	6.15 (dd, 15.1, 10.5 Hz, 1H)	131.5
10	6.07 (dd, 15.1, 10.7 Hz, 1H)	129.6
11	6.06 (dd, 15.3, 10.7 Hz, 1H)	132.9
12	5.71 (dd, 15.3, 7.4 Hz, 1H)	130.3
13	1.76 (d, 7.4 Hz, 1H)	18.4
14	0.89 (d, 7.2 Hz, 3H)	15.0
1-NH	2.48 (br, 1H)	
4-OH	2.48 (br, 1H)	

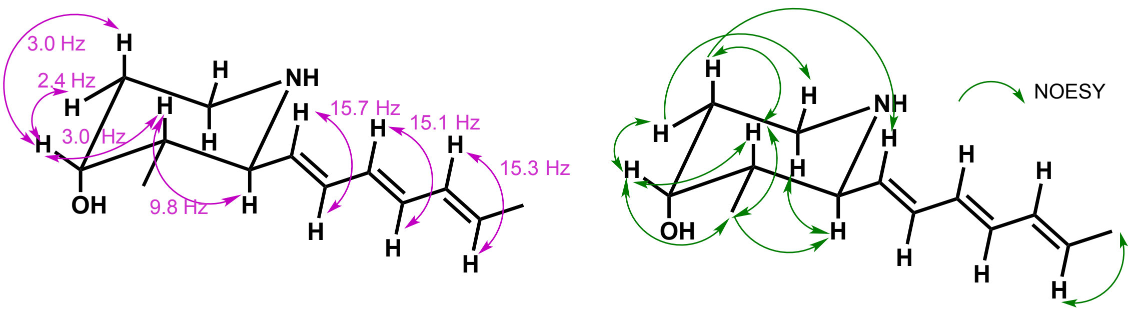

Figure S15. Coupling data and NOESY data for **7**.
